# MXenes Thin Films: From Fabrication to Their Applications

**DOI:** 10.3390/molecules27154925

**Published:** 2022-08-02

**Authors:** Israt Ali, Muhammad Faraz Ud Din, Zhi-Gang Gu

**Affiliations:** 1 Fujian Institute of Research on the Structure of Matter, Chinese Academy of Sciences, Fuzhou 350002, China; isratali@fjirsm.ac.cn; 2 University of Chinese Academy of Sciences, Beijing 100049, China; 3 Institute of Physics, Slovak Academy of Sciences, 84511 Bratislava, Slovakia; faraz.din@savba.sk

**Keywords:** MXenes, MXenes thin films, MXenes films fabrication methods, MXene films applications

## Abstract

Two-dimensional MXenes possessed exceptional physiochemical properties such as high electrical conductivity (20,000 Scm^−1^), flexibility, mechanical strength (570 MPa), and hydrophilic surface functionalities that have been widely explored for energy storage, sensing, and catalysis applications. Recently, the fabrication of MXenes thin films has attracted significant attention toward electronic devices and sensor applications. This review summarizes the exciting features of MXene thin film fabrication methods such as vacuum-assisted filtration (VAF), electrodeposition techniques, spin coating, spray coating, dip-coating methods, and other physical/chemical vapor deposition methods. Furthermore, a comparison between different methods available for synthesizing a variety of MXenes films was discussed in detail. This review further summarizes fundamental aspects and advances of MXenes thin films in solar cells, batteries, electromagnetic interference shielding, sensing, etc., to date. Finally, the challenges and opportunities in terms of future research, development, and applications of MXenes-based films are discussed. A comprehensive understanding of these competitive features and challenges shall provide guidelines and inspiration for further growth in MXenes-based functional thin films and contribute to the advances in MXenes technology.

## 1. Introduction

**Figure 1:**
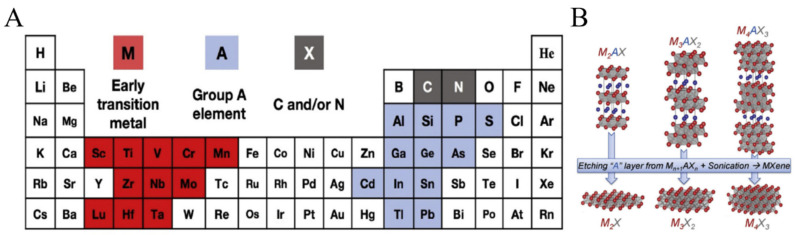
(**A**) Elements involved in the formation of MAX phase and (**B**) MXenes derived from different MAX phases. (reproduced with permission [4]. Copyright 2019, Elsevier).

The past decades have witnessed exceptional discoveries in the field of nanomaterials with extraordinary physical and chemical properties. Nanomaterials, regardless of their dimensions, have been recognized as one of the fascinating classes of nanoscience and nanotechnology in the past few decades. A tremendous amount of research work is devoted to the development of different dimensions containing nanomaterials because of their exceptional chemical, physical, electrical, optical, and magnetic properties [1]. Limiting to only one dimension, 2-D nanomaterials, a term that originated from “Graphene”, revolutionized the energy materials owing to extraordinary electrical and chemical properties [2,3]. Various other 2-D nanomaterials, including graphene, black phosphorous nanosheets, transition metal dichalcogenides, transition metal oxides, and molybdenum disulfides owing to unique 2-D structures, possess excellent electrical, chemical, and physical properties have been widely used in catalysis, energy storage, coatings, environment and in biomedical applications [4,5]. Recently, after graphene, MXenes emerged as a new class of 2-D materials in 2011 that can be obtained by etching and exfoliation of polycrystalline nanolaminates of ternary carbides and nitrides named MAX phases [6,7]. During this short time, different materials of MXenes have been used in plenty of applications, including catalysis, batteries, energy conservation, and biomedicine, because of their hydrophilic surfaces and higher conductivities (~20,000 S/cm^−1^) as compared to other 2-D materials including graphene [8,9]. Complied in the simplest formula M_n+1_X_n_, MXenes contain a transition metal, i.e., M in the formula (Zr, Ti, V, Hf, Sc, Nb, Cr, Ta, Mo), while X is carbon or nitrogen (Figure 1). Until now, almost 70 MXenes-based materials have been prepared while the increasing interest in these 2-D metal carbides and nitrides (MXenes) is growing exponentially [10].

Hexagonal layered MAX phase containing X atoms occupying octahedral sites, and A atoms acted as a bridge for M and X atoms attachment [11]. There are two featured bonding characters available in the MAX phase. One is related to covalent, ionic, and metallic bonding, i.e., expressed by the M-X bond, and the second is the M-A bond, which mostly corresponds to a metallic feature [12]. Because of such a strong bonding between the MAX layers, it is a complicated task to separate these layers from each other mechanically, unlike graphite and transition metal dichalcogenides (TMDs), where the featured bonding is weak van der Waal forces. MAX phase possesses not only the properties of ceramics, including thermal stability, suitable strength, and fragility but also conducting heat and electricity like metals [13]. To achieve single or multilayered MXenes, it is mandatory to selectively etch the A layer from the MAX phase. The obtained MXenes sheets have shown different properties compared to their parent precursors. There are three MXenes lattice structures one can obtain from MAX (i) M_2_X (ii) M_3_X_2_ (iii) M_4_X_3_ with 3, 5, and 7 atomic layers (Figure 1) [4].

Fabrication of thin films of different 2-D materials paved a new path in research development. A variety of thin film fabrication methods such as dip coating, spin coating, spray coating, chemical/physical vapor deposition, electrodynamic atomization, roll-to-roll, and magnetic assemblies are used, depending on the requirements [14]. Different film fabrication technologies have been employed for the fabrication of thin films of 2-D materials, including gold nanoparticles, graphene, carbon nanotubes, and black phosphorous, gained tremendous attention, but difficulty in thin film fabrication and unsatisfying stripping strategies limit their use for advanced applications [15,16,17]. MXenes owing to their exceptional surface, chemical, and physical properties, have also been employed for thin film fabrication.

Recently, plenty of review articles have explained the synthetic procedure, properties, and applications of MXenes. Still, it is hard to find a single review article covering all the literature available about thin films of MXenes. This detailed review article will focus on the complexities in the synthetic procedures of MXenes thin films. Several engineering methods, including vacuum filtration method, spin coating, spray coating, dip coating, electrodeposition, electrochemical polymerization, and spin-spray coating discussed in detail to understand the complexity of MXenes thin films fabrication. Further, a comprehensive comparison between the available fabrication methods defines the suitability of MXenes films for different applications. Finally, we summarize different advanced applications of MXenes films in electromagnetic interference shielding, photodetectors, solar cells, batteries, and separation.

## 2. Structure and Properties of MXenes

The etching of MXenes with different acids introduced the hydroxyl (OH)-, oxygen (O)-, and fluorine (F)- based functional moieties on the surface [18]. Synthetic strategies and the MAX phase are the most critical parameters that could control the functionalization of different groups on the surface of MXenes. The confirmation of the structural changes of MXenes has been performed by using X-ray crystal diffraction (XRD), X-ray photoelectron spectroscopy (XPS), and other electron microscopic techniques. The removal of aluminum (Al) by using acids has been confirmed by XRD analysis as the peak of Al from Ti_3_AlC_2_ etched away [19]. After sonication, the final structure also loses its crystalline nature and shows a decrease in peak intensity. Further confirmation of the presence of O, OH, and F functional groups has been confirmed by XPS analysis [20].

After plenty of computational studies, it has been confirmed that MXenes are classified into different structures based on the central metal atom, for example, monometallic MXenes Ti_2_C, Nb_4_C_3_, and bimetallic MXenes (Ti_2_−yNbyCTx) in which one transition metal occupied the external layer [6]. Chemical orders in MXenes are dependent on the nature of the respective MAX phase [21]. Two fundamental chemical orders exist in the MAX phase, (i) firstly, one or two layers of M element get sandwiched in between the layer of another M element, it is also known as out-of-plane order, and (ii) in-plane order, in these two M elements are ordered in the plane. MAX phase conversion to MXenes does not affect the chemical orders [22,23]. However, the pattern of the atoms depends on the number of metals present for bonding; for instance, in the case of monometallic, the M layer is composed of only a single type of transition metal, e.g., Ti_2_CT_x_, V_2_CT_x,_ and Ti_3_C_2_T_x_, where T_x_ is representing different functional groups (F, OH, O). The other class is bimetallic transition metal-containing MXenes, in which two transition metals are available for the formation of two metal layers. When two different transition metals are ordered in an alternative manner, different planes containing MXenes are formed. In the case of in-plane (M′_4/3_M″_2/3_XT_x_) ordered MXenes, each M layer atomic plane comprises two different transition metals that are attached in an alternating manner. While out of the plane (M′_2_M″X_2_T_x_) MXenes, one transition metal layer is sandwiched between two identical types of transition metals. Mostly the external layer is occupied by Mo and Cr, while other transition metals are present in the center, e.g., Mo_2_TiC_2_ and Mo_2_Ti_2_C_3_ [24]. In the case of solid solution MXenes ((M′,M″)n_+1_C_n_T_x_), all M layers contain two transition metals, and these two metals are randomly distributed, e.g., (Ti, V)_3_C_2_ and (Cr, V)_3_C_2_ [24]. The synthesis of double-transition metal-containing nitrides and carbonitrides is still not available. M atoms are arranged in a hexagonal fashion, and the octahedral sites are covered with X atoms.

In the case of electronic structure, MXenes possess metallic conductivity because of electronic density near to Fermi level [25]. The position of metals plays an essential role in the electronic properties of MXenes; for instance, in the case of double-transition metal MXenes the outer metal layer has shown a significant influence on the electronic performance [26]. Further, functionalities (O, F, and OH) imparted on the surface of MXenes after etching played a significant role in the electronic conductivity of MXenes [27]. In the case of oxygen termination, mostly the MXenes are semiconductors because oxygen can accept two electrons compared to F and OH terminated MXenes. The hybridization between the metal d-orbital and functional group p orbital significantly reduces the density of states, which may cause the lifting of d-orbital above the Fermi level. Hence, MXenes’ electronic structures depend on the metals, X atoms, and finally on their terminated functional groups [28].

Owing to its excellent electronic nature, it is crucial to understand the electronic mobilities in MXenes. The electronic properties of MXenes can be modified because of the presence of transition metals. Transition metal d-electrons mainly control the density of states present near the Fermi level. Therefore, the electronic properties can be tuned by surface terminating functional groups. Hence, metallic MXenes can be converted into semiconducting ones by simply modifying the surface functionalities. Based on electronic properties manipulation, MXenes are divided into two categories topologically trivial and non-trivial metallic/semimetallic or semiconducting MXenes [26,29].

The optical properties of MXenes are not very well explored. The absorbance of the MXene, either in the colloidal state or in thin films, depends on the amount of MXene in the solution. In the case of thin films, the absorbance increases with the thickness of MXene thin films. In the case of transmittance, it has been seen that Ti_3_C_2_T_x_ MXenes in pure and intercalated form obtained 77% and 90% transmittance in visible light with a wavelength of 550 nm [30]. The optical properties of MXenes depend on the functional groups (F, OH, and O) functionalizing on the surface. In-plane absorption coefficients are lower for F and OH functionalize Ti_3_C_2_ MXenes in infrared to the ultraviolet light range [31]. While in the case of bare and O functionalized MXenes, the in-plane absorption coefficients are higher [32].

The magnetic properties of MXenes can also be tuned by utilizing d-orbital electrons of transition metals. In most cases, spin-polarized density functional calculations indicated that most MXenes are nonmagnetic in the ground state because of the strong interaction between transition metal, X elements, and other chemical functionalities [33]. Hence, a variety of magnetism can be seen in different MXenes such as 2-D Cr_2_C, Cr_2_N, Ta_3_C_2_, and Cr_3_C_2_ are ferromagnetic in nature, while few of the 2-D titanium carbide and nitrides are antiferromagnetic [34,35]. It is also noteworthy that F, H, OH, and O containing Cr_2_C and Cr_2_N MXenes have shown magnetic, ferromagnetic, and antiferromagnetic properties because transition metal contains electrons in the d-orbital [36]. Mixed and Janus MXenes containing different metals and different functionalities have shown magnetic, antiferromagnetic, and ferromagnetic behavior under a small electric field [37,38]. Strain also has played a significant role in changing the magnetic properties of different MXenes by deforming the structure. Half metallic ferromagnetic Ti_2_C can be converted into a semiconductor and then into a metal under biaxial strain, while this conversion is not observed in Ti_2_N MXenes. Similarly, V_2_C and V_2_N have shown more significant magnetic moments under biaxial tensile and strain [39,40]. Hence, the electric and magnetic properties of MXenes are tunable, which leads to a diversity of applications of different MXenes in energy storage, supercapacitors, and sensors.

## 3. Synthesis of MXenes

MAX phase is the precursor for the synthesis of varieties of MXenes. Typically, a wet chemical etching method with a strong acid hydrogen fluoride (HF) has been employed to remove the A layer from the MAX phase to synthesize multilayered MXene flakes. The M-A bond in MXenes is metallic, and it is hard to break this bond with standard exfoliation strategies, unlike graphene. Synthetic approaches for MXenes are critical as they decide the size of MXenes and the nature of applications for prepared MXenes. Bottom-up and top-down methods have been used for the synthesis of MXenes. However, a bottom-up approach such as chemical vapor deposition (CVD) causes the formation of high-quality films of MXenes on different substrates; the shape of multilayered films limits their application for the synthesis of different MXenes. For instance, Xu et al. [41] utilized CVD to grow over 100 mm thick and high-quality Mo_2_C, tungsten carbide, tantalum carbide, and multilayered thin films. Similarly, Geng et al. [42] have used the CVD approach to grow uniform and high-quality Mo_2_C on the graphene substrate.

**Figure 2:**
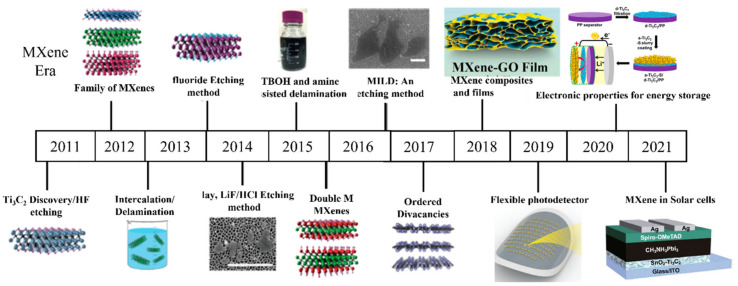
Development in the synthesis of MXenes (reproduced with permission [50]. Copyright 2021, Elsevier), (reproduced with permission [51]. Copyright 2019, Wiley-VCH), (reproduced with permission [52]. Copyright 2018, American Chemical Society), (reproduced with permission [53]. Copyright 2020, Wiley-VCH).

In the beginning, multilayered nanoflakes of different MXenes, including V_2_CT_x_, Nb_2_CT_x_, Ti_3_CNT_x_, and Ti_2_CT_x,_ were synthesized using the wet chemical method. Delamination of multilayered MXenes was not achieved successfully until 2013, when Y. Gogotsi et al. used organic solvent (DMSO) to delaminate Ti_3_C_2_, Ti_3_CN, and TiNbC, MXenes into single flakes with enhanced conductivity and photothermal properties [43]. Further, with the development of synthetic approaches, ammonium bifluoride (NH_4_HF_2_) salt has been used to etch the A layer [44]. To avoid the direct use of HF for the synthesis of MXenes because of its harmful and corrosive nature, in situ production of HF was introduced by the reaction of lithium fluoride (LiF) with hydrogen chloride (HCl) [45]. In addition, isopropylamine, tetrabutylammonium hydroxide (TBAOH), tetrapropylammonium hydroxide (TPAOH), [46,47], and tetramethylammonium hydroxide (TMAOH) have successfully been used for the delamination of layered MXene [48,49]. Minimally intensive layer delamination (MILD) was introduced for the manual delamination of the layered MXenes by just shaking without doing any sonication after acid treatment for the synthesis of larger but high-quality MXene flakes. The development era of the MXene materials can be seen in Figure 2.

Plenty of factors must be considered to successfully synthesize ultrafine MXene sheets, including the concentration of acids for the etching of the A layer, temperature, sonication, and centrifugation. MXenes are not very stable in the air or at high temperatures and can be oxidized easily. Recently, M. Malaki et al. [54] explained in detail the effect of sonication power, frequency, time, and modes on lateral size, morphology, and surface properties of MXenes. It is well noted that by using probe sonication along with bath sonication, there is an apparent reduction in the size of MXene sheets, and it is possible to break down the sheets to 50 to 150 nm in size by physical means (probe sonication and bath sonication) even without using some organic bases. Regarding sonication methods, it is necessary to manage the sonication time. MXenes cannot resist oxidation in the open air for a long time, and it imparts a significant effect on the physical and chemical properties. Probe and bath sonication of MXenes always required a lower temperature (4–10 °C). Experiments performed on the synthesis of MXenes showed that it is stable for up to 24 h but oxidizes after sonication for a long time. Similarly, L.Verger et al. [55] have explained the synthesis mechanism of MXenes by using different strategies such as the top-down method and bottom-up approach. A variety of etchants and intercalates, including HF, HCl, and fluoride salt that could cause the in situ formation of HF, NH_4_Cl, TMAOH, and TPAOH, have been studied in detail, and it always affects the size, morphology, and surface properties of MXenes.

Etching of MAX phase to MXenes is a hit and trial method. In most cases, Al is there for etching, and plenty of MXenes are formed by etching Al. Only 20% of the available MAX phases are etched to MXenes. In a recent study, Ti_3_C_2_T_x_ was obtained using the Al-free MAX phase, i.e., Ti_3_SiC_2_. However, it is well known that the etching of MAX phases without Al will not always result in the same MXenes as in the case of Ti_3_SiC_2_.

## 4. MXenes Thin Films

The increasing demand for nanomaterials forced researchers worldwide to focus on new synthetic routes and advanced applications. A wide range of nanomaterials has been discovered and utilized in energy storage, catalysis, biomedical field, and sensors. The response of individual nanomaterials has proven to be extraordinary, but the conversion of these nanomaterials into thin films provided a new direction to the nanomaterials field. Recently, 2-D monolayer films in the nanometer (nm) range thickness revolutionized research innovation. On the other hand, the precise regulation of precious metal nanoparticles assembled into an ordered 2-D or 3-D superstructure is an effective path to advanced functional materials and practical applications. Similarly, MXenes thin films have been synthesized by utilizing different methods, including vacuum filtration, interfacial assembly, electrochemical polymerization, spray coating, spin coating, dip coating, spin-spray coating, and physical vapor deposition (PVD).

### 4.1. Vacuum Filtration Method

**Figure 3:**
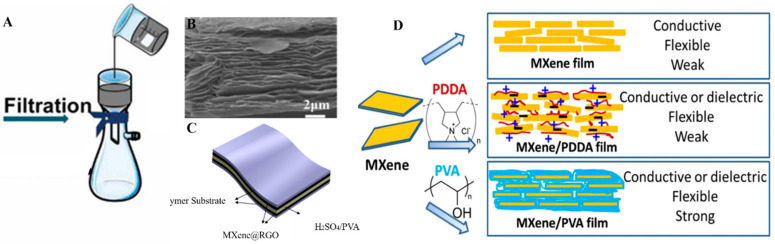
(**A**) VAF method for MXene thin films (reproduced with permission [56]. Copyright 2021, Elsevier); (**B**) SEM image of a multilayered thin film of Ti_3_C_2_ sheets (reproduced with permission [68]. Copyright 2018, American Chemical Society); (**C**) Application in supercapacitors, (reproduced with permission [62]. Copyright 2020, American Chemical Society); (**D**) Adjusting the properties of MXenes with different polymers (reproduced with permission [67]. Copyright 2014, National Academy of Sciences).

Uniform and well-controlled MXene thin films are required for different applications, including energy storage, conversion, and optoelectronic applications. The simplest method to achieve uniform MXene thin films is Vacuum-assisted filtration (VAF). In this method, a stable dispersion of MXene was prepared, and a suction filtration device was used to filter it. MXene sticks on the porous membrane’s surface after filtration and forms a thick layer (Figure 3A) [56]. The thickness of the film depends on the concentration of MXene, and as filtration is a self-limiting phenomenon, so uniformity of the film on the filter membrane is self-leveling [57] van der wall forces are also involved in bringing the MXenes closer to each other on the surface of the filter membrane. After that thick MXene layer need to be peeled off from the filter membranes and can be used in different applications. Filter membrane, suction speed, solvent, and MXene concentration are the factors that affect film formation. Although the VAF method is easy to use, it takes much time and energy to prepare a film. To make this process fast, a different approach is used by adding a small amount of alkali in Ti_3_C_2_ MXene before filtration to narrow down the time of filtration to about dozens of seconds rather than hours [58]. Another critical aspect of the MXene thin films fabricated by the VAF method is the peeling of complete film for patterning it to obtain advanced applications. Obtaining a thick layer from VAF could lead to ease in peeling from the filter membrane, and then patterned cutting of this film through laser enhanced its applicability in tattoo-based sensors and electromagnetic interference shielding [59,60].

Hu et al. fabricated a thick film of few-layered Ti_3_C_2_ MXene nanosheets using the vacuum deposition method on Millipore filters [61]. The uniformity of the MXenes thin films fabricated by using the VAF method has been confirmed by utilizing electron microscopy (Figure 3B). Plenty of MXene composites-based thin films were prepared by using the VAF method. To provide unique properties to MXenes thin films, polymers were functionalized on the surface by chemical and physical means. For instance, conductive polymer polyvinyl alcohol (PVA) mixed with Ti_3_C_2_ to form flexible thin films by exploiting the VAF method for high-capacity charge storage applications (Figure 3C) [62]. MXene composite materials in thin films have been used in various applications, including solar cells, batteries, supercapacitors, adsorbents, and energy conversion systems [63]. The mandatory requirement to utilize the properties of MXenes in solar cells, batteries, supercapacitors, and adsorbents is to obtain its uniform thin film. In this regard, Huang et al. designed a highly stable, flexible, and higher specific capacity (221 Fg^−1^) containing MXene@carbon nanotubes and MnO_2_-based hybrid thin film via the VAF method to use it as a supercapacitor [64]. Further, to obtain insight into the advanced level studies of MXene-based thin films for supercapacitors, the VAF method was utilized to fabricate MXene@Holey-Graphene films. Holey graphene was used to avoid self-restacking of Ti_3_C_2_, and a very unique, highly porous connectivity network was established there for the immense acceleration of ion transport, and it also shortened the transport pathway for ions and electrons [65]. VAF method has been used to fabricate micro-supercapacitors of Ti_3_C_2_ MXene. Well-dispersed MXene solution was vacuum filtered on an interdigital patterned mask and obtained a thin, highly conductive film used as a supercapacitor [66]. Other composites also cause a significant impact on the physical properties of MXenes. The nature of the dopant or polymer decides the final product’s flexibility, conductivity, and chemical properties. To tune the mechanical properties of MXenes, Ling et al. tried poly(diallyldimethylammonium chloride) and PVA polymers with MXene, and the resulting thin films have shown different properties in terms of conductivity and flexibility (Figure 3D) [67].

Tuning of the thickness of MXene film is required to use it in different electronic applications. Lightweight and ultrathin MXene films are needed for integrated electronics. Composite of MXene sheets with conductive polymers are not always favorable for energy storage and electronic applications. A controlled amount of dopant is required to obtain an advantage from the MXene composite films. For instance, to avoid electromagnetic waves, Liu et al. designed a Ti_3_C_2_/PEDOT: PSS-based biomimetic thin films shield by using VAF [68]. VFM has also been utilized to form mixed metal-containing MXene materials. In this regard, Halim et al. fabricated free-standing Mo_2_TiC_2_T_x_ MXene thin films by simply using the VAF method [69]. Similarly, doping into MXene leads to improvement in the mechanical properties of the MXene films. Dopamine doping in Ti_3_C_2_ MXene thin films provided seven times enhanced tensile strength with a tremendous increase in elongation fabricated by VAF [70]. 

### 4.2. Electrodynamic Atomization and Electrophoretic Deposition

**Figure 4:**
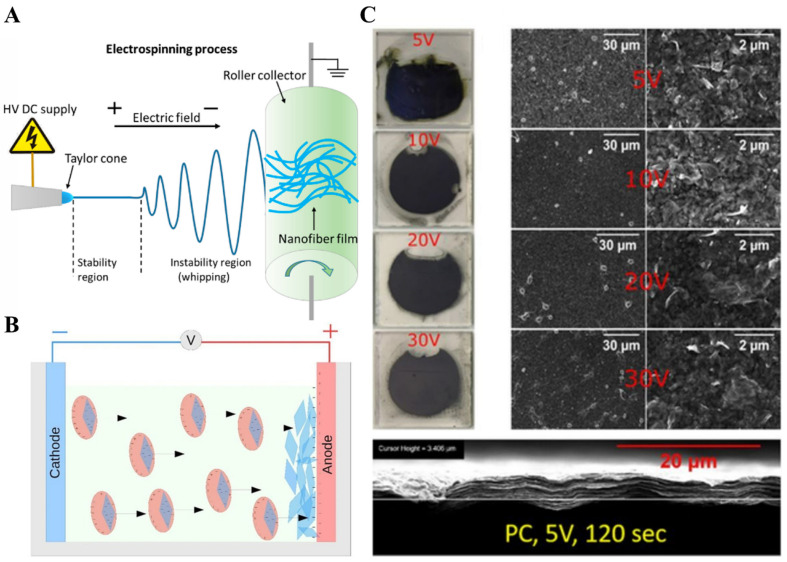
(**A**) Electrospray; (**B**) Electrophoretic method for the fabrication of Ti_3_C_2_T_x_ films; and (**C**) SEM images of the Ti_3_C_2_T_x_ formed under different voltage conditions for 600 sec each in water and polypropylene carbonate suspension. (reproduced with permission [74]. Copyright 2021, MDPI) (reproduced with permission [75]. Copyright 2017, IOP Science).

The electrohydrodynamic atomization technique (EHAT) has been utilized to form thin films of different materials. EHAT can be performed in two ways, (i) electrospraying and (ii) electrospinning [71]. In a typical EHAT process, the formation of droplets occurs when a well-dispersed liquid is poured into the nozzle. On applying a strong electric field, these droplets get charged, and the droplet at the end of the nozzle forms a cone shape spraying mode. A charged jet of liquid fall on the collector, which has an opposite charge to droplet or grounded. By changing the voltage, and distance between collector and droplet spray, this technique causes the formation of nanoparticles or fibers [72]. Different atomization modes can be applied to form particles and fibers, including micro-dripping, spindle, cone-jet, and multi-jet. Droplet size depends on the applied voltage, flow rate, and physical properties of the liquid. In the case of MXenes, Ali et al. utilized the EHAT technique for the hole-free and uniform growth of Ti_3_C_2_Tx (100–400 nm) MXene thin film [73]. Similarly, Fang et al. applied the electrostatic and electrospray deposition method to fabric MXene/polymer-based composite thin films and have been utilized for strain sensors (Figure 4A) [74].

While in the case of electrophoretic deposition (EPD), an electric field is applied to the stable colloidal suspension present between two electrodes, and it causes the deposition of charged MXenes on the respective electrode (electrophoresis). In the second case, after applying an electric field to the colloidal solution containing MXenes, deposit coagulation is formed at the interface of suspension and electrode (deposition) (Figure 4B) [75]. On varying the voltage, the film formation process, thickness, morphology, and flake orientation may change. To test this, Collini et al. fabricated Ti_3_C_2_ MXene thin films by EPD process at 5 V, 10 V, 15 V, and 20 V for 600 s each, and it was observed that initially, the conductivity of Ti_3_C_2_ MXene thin films was decreased, but later for 10 V, it increased to a specific value (Figure 4C).

### 4.3. Spin Coating Method (SCM)

**Figure 5:**
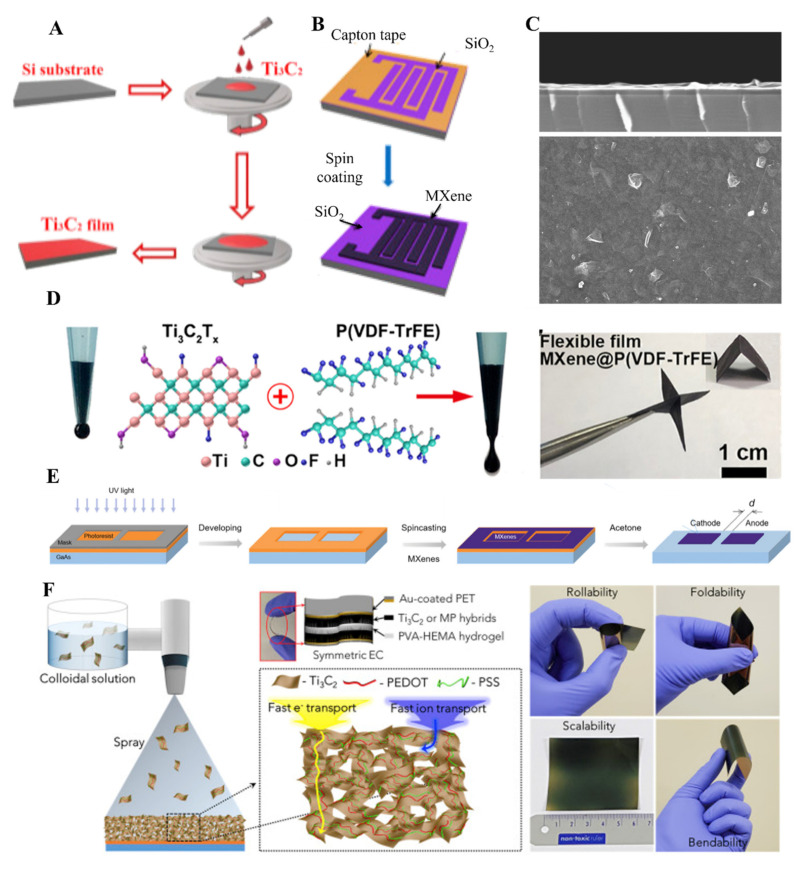
(**A**) Schematic drawing of SCM (reproduced with permission [78]. Copyright 2019, Elsevier); (**B**) Schematics of Ti_3_C_2_T_x_ MXene film formation on patterned silicon substrate using SCM method, (reproduced with permission [79]. Copyright 2020, Elsevier); (**C**) SEM images of Ti_3_C_2_T_x_ MXene film formed by SCM. The scale bar is 5 µm (reproduced with permission [80]. Copyright 2019, Wiley-VCH); (**D**) Fabrication of Ti_3_C_2_T_x_ MXene film on a flexible substrate with SCM (reproduced with permission [85]. Copyright 2020, American Chemical Society); (**E**) Photolithography was performed for patterning, and SCM was utilized for the fabrication of MXene film on the designed surface (reproduced with permission [86]. Copyright 2019, Wiley-VCH); (**F**) Schematic of MXene@PEDOT: PSS thin films fabricated using SCM and symmetric capacitors, photographic images of composite MXene thin films their scalability, foldability, and rollability (reproduced with permission [83]. Copyright 2018, Elsevier).

Spin coating is a standard method for fabricating thin films of different materials [76,77]. In this technique, a substrate is spun at high speed with a liquid solution on its surf. The involvement of centripetal force and surface tension of the liquid cause even distribution of the liquid on the surface of the substrate. After evaporating any solvent from the surface of the substrate, a few nanometers to micrometers of thin films can be obtained. MXene thin films were also fabricated by using the spin coating method (SCM). The nature of the substrate is dependent on the application of the MXene materials. For instance, for solar cell fabrication ITO, FTO, and other flexible substrates were used for spin coating of MXenes. The spin coating can be performed by casting a solution on a spinning substrate or pouring a solution onto a stationary substrate and rotating it to the required rotation to obtain thin films. A substrate with a 10 cm diameter can be used commercially in spin coater. Two significant factors that affect the formation of MXene films in SCM involved rotation speed and the concentration of MXene in a respective solvent. Higher the rotation per minute, less area will be covered by the film on the substrate surface. Similarly, a high concentration of the MXene solution will cover the larger area on the substrate, and a uniform film will obtain. In contrast, a gradual increase in speed will cause a decrease in the thickness of the MXene film. To obtain a uniform thin layer, it is mandatory to reach the ramping speed in SCM. The multilayered thin films can also be obtained with SCM. By changing the parameters mentioned above, it is possible to change the thickness of the MXene films in SCM. In this regard, Zhang et al. fabricated Ti_3_C_2_ MXene thin films on silica substrate by using SCM for a memristive device (Figure 5A) [78]. The film’s characterization was performed using different microscopic techniques, including SEM, TEM, and AFM, and the thickness of the Ti_3_C_2_ film was about 2.2 µm. It is possible to design intelligent thin films by using SCM. SCM has also been used in fabricating MXene thin films on patterned substrates. For instance, Huang et al. fabricated Kapton masked hydrophilic silica substrate to obtain suitable adhesion of Ti_3_C_2_ MXene thin films (Figure 5B) [79]. The treatment of silica substrate with hydrophilic groups causes uniformity and integrity in the thin film Ti_3_C_2,_ while the thickness of the film was about 142 nm. Ti_3_C_2_T_x_ MXenes synthesized by SCM have shown excellent physical, electronic, and chemical properties as it is easy to tune the thickness. Dillon et al. fabricated highly conductive Ti_3_C_2_ MXene films with a roughness of 3 to 9 nm and tunable thickness ranging from 2 to 81 nm (Figure 5C) [80]. Different titanium carbide films synthesized using SCM have shown the highest conductivity value of about 5000 S cm^−1^. The transparency of these films was about 80% [30,81]. SCM is not only limited to Ti_3_C_2_ and Ti_2_C MXenes but has been used for the fabrication of other MXenes. Ying et al. have used SCM to fabricate transparent V_2_CT_x_ films in tetrabutylammonium hydroxide (TBAOH) dispersant. The conductivity value for thicker and thinner V_2_CT_x_ films was about 3300 ± 100 S/cm [82]. Because of the improved electrochemical performances of the MXene thin films fabricated by using SCM, this method has been explored to fabricate MXene-based composite films. MXene-based composite thin films have also been used in different applications, including energy storage and sensors, which require foldability, flexibility, and scalability. In this regard, Gund et al. fabricated Ti_3_C_2_@PEDOT: PSS-based flexible and foldable thin films to be used as supercapacitors. The thickness of the film obtained was about <1 µm, and it has a faster charge and ion transportation (Figure 5F) [83].

The number of layers of MXene sheets could also be tuned by repeating the spin cycles. By increasing the speed of the rotator and diluting the solution to a more considerable extent, it is possible to attain single-layered MXene thin films for a variety of applications. For instance, Zou et al. fabricated the thin films of Ti_3_C_2_ at a lower rotation speed of about 200 rpm, and the thickness of the film was about 25 µm [84]. Another important aspect of SCM is the fabrication of thin films on different substrates, including glass, ITO, FTO, and polymeric flexible substrates. To obtain free-standing and flexible substrate-based MXene nanosheets, Li et al. have utilized SCM to fabricate Ti_3_C_2_ and poly(vinylidene fluoride) trifluoroethylene composite MXene thin film on a glass substrate. After getting a uniform thin film on a glass substrate, it was heated to about 80 °C and then immersed in water to obtain free-standing flexible MXene thin films for wearable electronics (Figure 5D) [85]. Patterned MXene thin films on different substrates were obtained by using complicated techniques involving laser cutting and printing techniques. SCM has also been utilized to fabricate patterned thin films of MXene, but it involves the masking of the substrate. In this regard, Montazeri et al. masked the gallium arsenide (GaAs) substrate for the fabrication of guided and patterned Ti_3_C_2_ thin film (19.2 nm) to be used in photodetector (Figure 5E) [86]. Hence, by utilizing SCM, it is possible to coat different MXene materials on various surfaces, either solid or flexible, for useful applications. MXene thin films fabricated by SCM are homogeneous in nature because of the involvement of different forces, including electrostatic, centrifugal, air shear, and viscous forces. These forces cause the adsorption and rearrangement of the MXene thin films along with dehydration of the films on the substrate [87]. In summary, SCM is a widely used method for fabricating single-layered or multilayered uniform, homogeneous and thin MXene films.

### 4.4. Spray and Dipping Method

**Figure 6:**
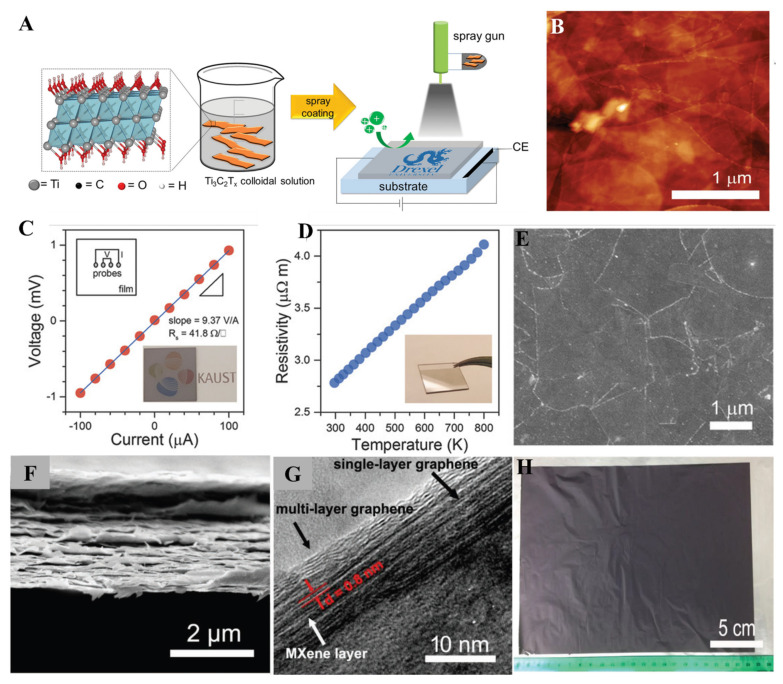
(**A**) Spray coating of Ti_3_C_2_ MXene; (**B**) AFM image of the MXene thin film synthesized by using the spray-coating method, (reproduced with permission [88], Copyright 2016 Wiley-VCH); (**C**–**E**) I-V curves, resistance vs. temperature plot, and SEM image of the Ti_3_C_2_T_x_ thin film fabricated by using the spray-coating method, (reproduced with permission [89]. Copyright 2018 Wiley-VCH); (**F**–**H**) Cross-sectional SEM image, high-resolution TEM image, and photographic picture of the MXene@rGO thin films (reproduced with permission [90]. Copyright 2019 Wiley-VCH).

Defect-free films are essential for higher conductivity values. The Chemical Vapor deposition method has been used to form various films on different substrates. The higher fabrication cost and limitations in size cause commercialization problems. Further, spin coating and electrodeposition methods have shown higher R_s_ values because of inter-flake distance and defects. The spray-coating method emerged as a new solution-based method for fabricating defect-free MXene films to overcome these obstacles. The spray-coating method is the fastest method for the fabrication of thin films, and in comparison to SCM, it is helpful on an industrial scale. Consecutive washing and spraying MXene solution on the substrate can cause the fabrication of thin and uniform layers. It is beneficial to control the thickness and transparency of MXene thin films. Spray flow, spray speed, nature of substrate, and cycles of spray affect the viscosity of MXene thin films. For instance, Gogotsi et al. fabricated Ti_3_C_2_ MXene thin film with thickness varying from 5 to 70 nm by utilizing the spray-coating method (Figure 6A) [88].

The spray-coating method is considered the best option for large area processing techniques, including inkjet printing and roll-to-roll processing. In the case of device fabrication, researchers always require a suitable method to obtain excellent electronic and chemical properties from the fabricated MXene thin films on a large scale. The spray-coating method has also been explored to fabricate large-scale MXene thin films without causing any damage to their physical or chemical properties. Wang et al. synthesized large area Ti_3_C_2_ MXene thin films for the n-type and p-type transistors with a thickness in the range of 3.69 nm and excellent electronic properties of MXene thin films obtained by using the spray-coating method (Figure 6B–E) [89]. MXene composite materials played an essential role in the enhancement of conductivity and improvement in the MXene electrochemical properties. The fabrication of large-scale uniform MXene thin films is a time-consuming process. The spray-coating method has been efficiently used to fabricate large-scale MXene film in a short time. Zhao et al. fabricated MXene/reduced graphene (r-GO) paper-based thin films by spray-coating method to prepare binder-free electrodes directly used in Na-ion batteries as an anode material [90]. Layer-by-layer assembly of Ti_3_C_2_ MXene and graphene films obtained by this method can be seen clearly from high-resolution transmission electron microscopy (TEM) (Figure 6F–H). In summary, the spray-coating method is widely used to fabricate uniform, ultrathin and flexible monolayer and multilayers films of MXene materials. In the spray-coating method, MXene layers are well organized and distinct from each other. 

**Figure 7:**
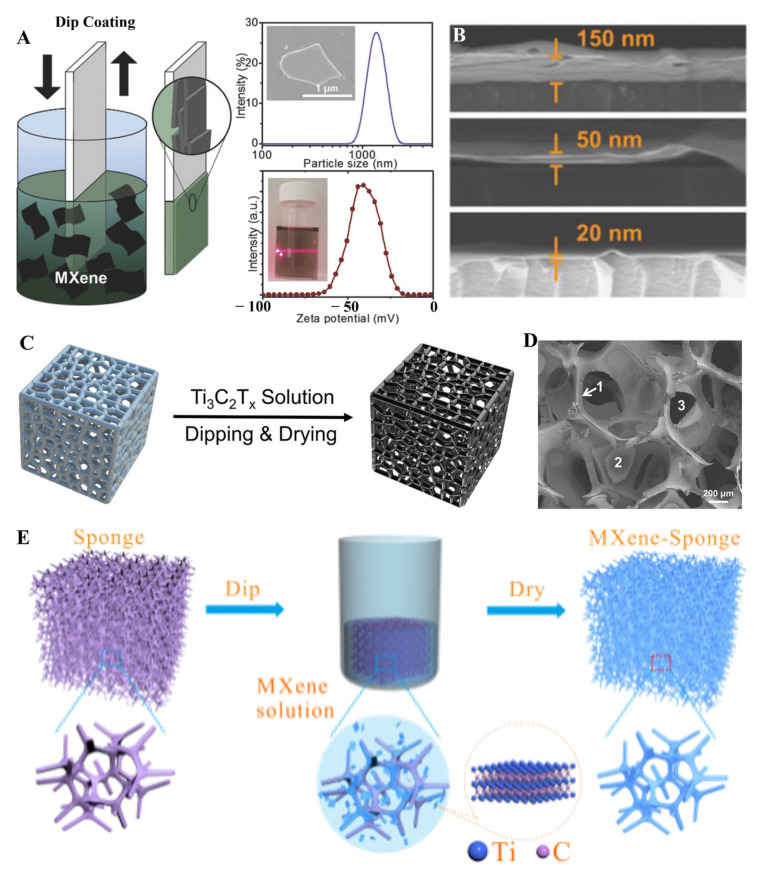
(**A**) Schematic illustration of the preparation of Ti_3_C_2_ MXene thin film by using the dip-coating method, DLS measurements including size and zeta potential value of as obtained MXene flakes; and (**B**) SEM images of Ti_3_C_2_T_x_ thin films with different thickness (reproduced with permission [93]. Copyright 2018 Wiley-VCH); (**C**,**D**) Schematic of coating MXene on sponge and SEM images of MXene sponge foam (1–3) showing the different Ti_3_C_2_ flakes, (reproduced with permission [91] Copyright 2020 Wiley-VCH); (**E**) Schematic process for the fabrication of MXene-based sponge, (reproduced with permission [96] Copyright 2018 Elsevier).

Different methods are required to tune the thickness of MXene thin films. It is always a requirement to improve the mechanical, chemical, physical, and electrical properties of MXene thin films. Although vacuum filtration, spray, and SCM proved efficient for fabricating transparent, highly conductive, and flexible thin films of MXenes. Still, due to ease in use and fabrication of ultrathin films with lower solution concentration, the dip-coating approach gained much attention. The dip-coating method (DCM) is another way to produce MXene thin films with tunable thickness and lower roughness. In this method, the substrate is manually immersed in the MXene solution to obtain thin films with different thicknesses. Different factors that affect the film fabrication process in DCM involve substrate nature, surface modification of substrate, dipping time, and concentration of MXene solution. For sufficient layer deposition, it is always required to dip the substrate in MXene solution for almost 5 min. Consecutive cycles of dipping and washing the substrate are required to obtain uniform and defect-free films. Due to less contact with open air, DCM provides a coating shield to MXene. MXene thin films prepared from DCM have been used in different applications, including electromagnetic interference shielding, micro-supercapacitors, flame retardancy, and a suitable absorber [91,92,93,94]. In the DCM, a substrate with or without functionalization is dipped into the solution containing MXene. After plenty of dipping cycles in and out, a very uniform thin layer formed on different substrates. Salles et al. fabricated Ti_3_C_2_ MXene thin film by utilizing the dip-coating method with extraordinary optoelectronic properties. The dip-coating method was employed to obtain the different thicknesses of Ti_3_C_2_ MXene thin films ranging from 20 to 150 nm (Figure 7A,B) [93]. Salles et al. fabricated another Ti_3_C_2_T_x_ MXene thin film with 30 nm thickness, different film surface roughness, and T550 nm = 65% transmittance to obtain a variety of applications [95]. Multiple factors are involved in getting a homogeneous and uniform layer of MXene in the case of DCM, such as dipping speed, MXene composition, substrate, surface chemistry of MXenes, and environmental factors. Higher the thickness of the films better the absorption in the UV region. It is always interesting to obtain a uniform assembly of MXene nanosheets on different sizes and shapes of the substrate. DCM proved to be an efficient method to obtain films of MXene on any kind of substrate, whether it is flat or randomly designed surfaces. In this regard, Shui et al. fabricated uniform Ti_3_C_2_ coatings on the sponge by simply dipping a sponge in Ti_3_C_2_ solution for about 5 min and acted as a suitable terahertz absorber (Figure 7C,D) [91]. Such versatility of DCM proved beneficial not only for energy storage devices but also for sensing devices. Such coatings are beneficial for different sensors because of the network-based structure of the sponge or coating material [96]. Similarly, Yue et al. utilized the 3D network of sponge material and coated it with Ti_3_C_2_ to obtain a piezoresistive sensor (Figure 7E) [96]. MXene-coated sponge material has shown excellent mechanical stability, porosity, and electrical properties. Controlling the number of layers of MXene thin films is another challenging task to improve the mechanical and chemical properties of MXene. The DCM proved to be an efficient method for controlling the number of layers of MXene sheets.

Bilayer Ti_3_C_2_@montmorillonite MXene sheets were fabricated by dipping the substrate into piranha solution for one hour and then in poly(vinylpyrrolidone) solution for 15 min. Lastly, dipping this substrate into Ti_3_C_2_@montmorillonite solution caused the formation of bilayer sheets. After repeating these dipping cycles, it is possible to obtain the desired number of bilayers of sheets [97]. Due to ease of use and inherent scalability, a drop-casting method is an excellent option for synthesizing large-scale MXene films. The drop-casting process has not been studied extensively because of planar and hydrophilic substrates. Despite such limitations, Lipton et al. fabricated large-scale (125 cm^2^) Ti_3_C_2_ MXene film with 23.2 µm thickness and 14 nm roughness [98]. Except for Ti_3_C_2_, other MXene materials, owing to their higher conductivity and excellent stability, were utilized to fabricate thin films to obtain advanced applications, including supercapacitors, sensors, and batteries. In this regard, Lee et al. fabricated vanadium carbide (V_2_C) thin films by using the drop-casting method for the gas-sensing application [99]. 

Surface modification of substrate plays an essential role in the uniform fabrication of MXene thin films. Plenty of substrates are treated with ozone prior to MXene film formation, but in some cases, to obtain uniform and strong binding of MXene with the substrate, its modification with hydrophilic or hydrophobic groups is considered to be an important step. In this regard, park et al. used flexible poly (ethylene terephthalate) (PET) substrate, modified its surface with amine groups, and then dipped it in the solution of MXene [100]. Uniform and well distribution of MXene thin films over all the surface of PET fibers were obtained only because of substrate modification. 

### 4.5. Other Methods

**Figure 8:**
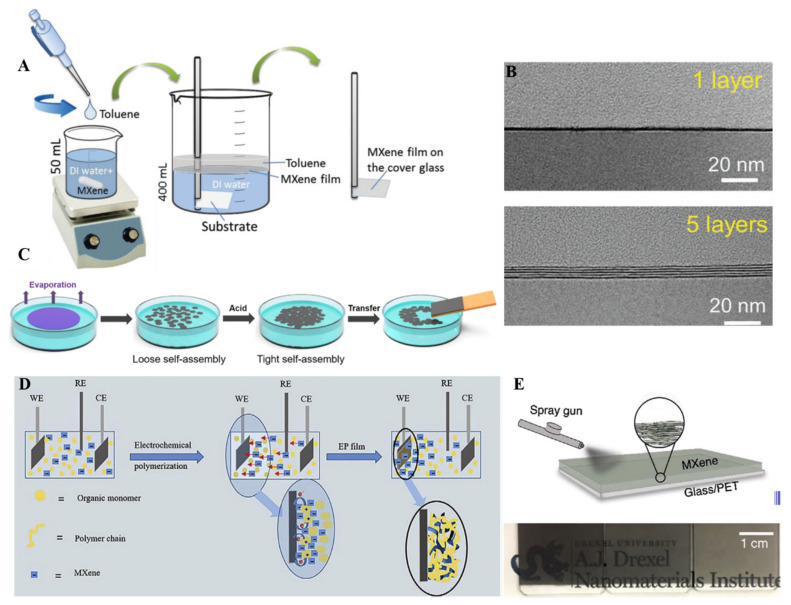
(**A**) Interfacial self-assembly method used for the fabrication of Ti_3_C_2_ MXene thin film with varied thicknesses (reproduced with permission [101]. Copyright 2018, Wiley-VCH); (**B**) Cross-sectional TEM images of the monolayer by monolayer, one layer, and five layers Ti_3_C_2_ MXene thin films fabricated by interfacial self-assembly technique (reproduced with permission [103]. Copyright 2019, American Chemical Society); (**C**) The interfacial assembly used for tightness of Ti_3_C_2_ MXene films (reproduced with permission [104]. Copyright 2020, Wiley-VCH); (**D**) Electrochemical polymerization process used to fabricate Ti_3_C_2_ MXene films (reproduced with permission [105]. Copyright 2019, Elsevier); (**E**) Spray gun-based method for the Ti_3_C_2_ film fabrication (reproduced with permission [106]. Copyright 2019, Elsevier).

Few other methods have also been used to fabricate MXene thin films with tunable thickness. Among them, the liquid–liquid interface method has been utilized for the fabrication of MXene thin films. In this method, two immiscible liquids were used to fabricate the thin film at the interface of these two liquids. In most cases, organic solvent (hexane, toluene) and inorganic solvent (water) have been used, and the slow evaporation of organic solvent causes the assembly of different nanomaterials at the interface. In this regard, Dong et al. fabricated Ti_3_C_2_ MXene thin films of different thicknesses (5–100 nm) by utilizing the interfacial film formation process (Figure 8A) [101]. Interfacial film formation is not an easy method compared to spin, spray or dip-coating methods. The rate of solvent evaporation and concentration of MXene are the key factors in controlling the film formation process at the interface. In another report, Chertopalov et al. synthesized less than 60 nm thick Ti_3_C_2_ MXene film to fabricate a Ti_3_C_2_-TiO_2_ composite that could change the electrical resistance under UV irradiation [102]. Another vital advantage of the interfacial thin film is the transfer of fabricated film to any substrate: solid glass, paper, or flexible curvy substrate. The interfacial film formation method is feasible to transfer thin films of MXenes to any substrate, and this method helps prepare ultrathin films of MXene with suitable transparency. Kim et al. utilized a liquid–liquid interface assembly of Ti_3_C_2_ with a thickness of 10 nm (Figure 8C) [103]. A high stacking order and vital plane to plane adherence with low resistance (310 Ω) film were obtained. Firstly, on evaporation of the organic solvent, the loose assembly of MXene sheets was obtained, but after the addition of HCl, the electrostatic repulsions between the flakes were broken, and tight self-assembly was observed. One of the challenging tasks in thin film fabrication is to control the layers. It is still hard to attain monolayer by monolayer MXene thin film fabrication by utilizing spray, spin, vacuum, and dip-coating methods. The interfacial assembly method was successfully employed to obtain monolayer by monolayer film of Ti_3_C_2_ MXene thin film by Yun et al. (Figure 8B) [104].

Due to complexity, the electrochemical polymerization process for the fabrication of MXene thin films is rarely explored. In this process, three-electrode systems were used to fabricate MXene thin film in the presence of different electrolytes. During electrochemical polymerization, electrolytes play a very critical role in providing a conductive environment and efficient doping in polymer film as a counter ion without participating in the chemical reaction. In this conductive environment, negatively charged Ti_3_C_2_ or Mo_1.33_C MXene made a stable suspension with monomer (pyrrole, EDOT). These monomers will lose electrons during the electrochemical polymerization process and form cationic radicals to form polymer chains. Negatively charged MXene will move toward the working electrode because of the electric field gradient and doped into the polymer chains to create a complex film (Figure 8D) [105]. Directly spraying the solution on the cleaned substrate is another method for fabricating MXene thin films. In this method, MXene solution was directly sprayed on the desired substrate with a spray gun, and the thickness of the film obtained was about 40 nm with 55% transmittance at 550 nm (Figure 8E) [106].

Another difficult, lengthy and complicated process for fabricating thin films of MXene is layer-by-layer assembly. It involved sequential nanometer-thick monolayers adsorption of oppositely charged compounds. Layer-by-layer formation of thin films can be obtained by dipping, spraying, or spinning, but Weng et al. designed a strategy in which spin and spray coating are utilized in a single process to obtain thin films of MXenes by using poly (vinyl alcohol) (PVA) and poly (sodium 4-styrene sulfonate) (PSS) [107]. The tuning of thickness, roughness, transparency, and conductivities was brought by using the spin-spray layer-by-layer method. Spin speed, spray time, and concentration of MXene are the critical factors in tuning the thickness and conductivity of the films.

In the physical vapor deposition method, materials first converted into vapor form are deposited on the solid substrate. Only a single study covered the indirect formation of Ti_2_C and Nb_2_C MXene thin film fabrication by chemical etching of magnetron sputtered Ti_2_AlC and Nb_2_AlC [108]. Firstly, Ti and C targets were ignited at a high temperature (810 °C) to form TiC, and then Ti, Al, and C were ignited at the same temperature to form Ti_2_AlC film on a sapphire substrate. The same procedure was repeated for the fabrication of the Nb_2_AlC film. LiF was used on these substrates for selective etching of Al to form Ti_2_C and Nb_2_C MXenes. Ti_3_C_2_ is a widely studied MXene material, but it is mandatory to check other MXene materials such as vanadium carbide, Molybdenum carbide owing to exceptional conductivity, low toxicity, cost-effectiveness, and higher stability. For instance, Wang et al. fabricated uniform molybdenum carbide (Mo_2_C) thin film using the magnetron-sputtering technique [109]. Thin films of Mo_2_C acted as an outstanding saturable absorber with significant modulation of 10.39% and 8.89% at 1064 nm and 1342 nm, respectively. The thickness of the films obtained by this method was about 4.3 nm to 4.7 nm. In summary (Table 1), different assembly technologies have been used to fabricate the MXene films with various thicknesses and roughnesses. MXene is an emerging material and still needs improvement in large-scale film fabrication with fewer possible defects. These methods are utilized at a lab scale for the fabrication of MXene films for a variety of applications, but to achieve industrial-scale applications still, more fabrication technologies need to be explored.

## 5. Comparison of Different Methods Involved in the Fabrication of MXene Thin Films

**Figure 9:**
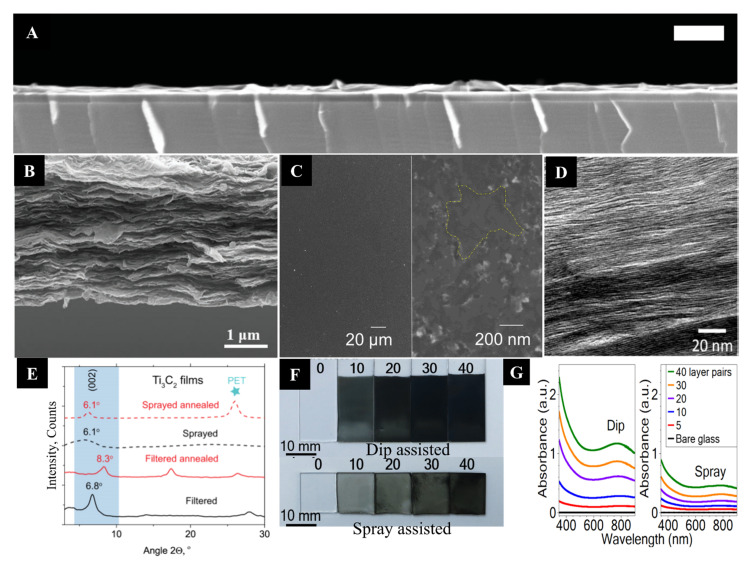
SEM images of (**A**) Ti_3_C_2_ thin film prepared by using SCM Scale (1 μm) (reproduced with permission [80]. Copyright 2016, Wiley-VCH); (**B**) The vacuum filtration method assisted the formation of Ti_3_C_2_ thin film (reproduced with permission [80]. Copyright 2016, Wiley-VCH); (**C**) Cross-sectional images of Ti_3_C_2_ thin film synthesized by using the spray-coating method (reproduced with permission [88]. Copyright 2016, Wiley-VCH); (**D**) SEM image of drop cast V_2_C MXene film, (reproduced with permission [99]. Copyright 2019, American Chemical Society); (**E**) X-ray diffraction (XRD) patterns of Ti_3_C_2_ MXene thin films prepared by VAF (solid black line) and after treatment in vacuum at 150 °C (solid red line) and spray coating of Ti_3_C_2_ film (black dashed line), and heated in film in vacuum at 150 °C (red dashed line); (**F**) Physical images of dip and spray assembled multilayer Ti_3_C_2_ coatings of a different number of layer pairs on a glass substrate, (reproduced with permission [140]. Copyright 2018, Wiley-VCH); (**G**) UV-vis absorption spectra of Ti_3_C_2_ at 770 nm wavelength vs. the number of layer pairs on a glass substrate (reproduced with permission [141]. Copyright 2018, American Association for the Advancement of Science).

Different methods involved in synthesizing MXene films are helpful for various applications. The SCM has been utilized to fabricate Ti_3_C_2_ MXene thin film for solar cells. In the case of batteries, vacuum filtration proved to be an efficient method. Further, layer-by-layer synthesis of MXene thin films is advantageous for electrochemical sensors, batteries, and electrode materials. The primary purpose of these methods is to tune the thickness of the MXene films from micrometers to nanometers for different energy storage and sensing applications. Although every technique helps synthesize a variety of MXenes with different thicknesses, a specific method is still required for some particular applications, as mentioned above. As to obtain a higher conductivity value, Dillon et al. have utilized the SCM for the fabrication of Ti_3_C_2_ MXene thin film (2 to 81 nm) with very low roughness (3 to 9 nm) (Figure 9A) [80]. Further, the controlled alignment of MXene sheets during film formation was achieved using the vacuum filtration method. It is a highly demanded method where thick MXene films are required (Figure 9B) [65]. Similarly, 5–70 nm and 20–150 nm thick Ti_3_C_2_ films were produced using spray and dip-coating techniques with suitable transparency, conductivity, and flexibility (Figure 9C) [88]. Although different methods have been used to fabricate MXene thin films and Ti_3_C_2_ is a widely studied MXene material, among other MXene materials, V_2_C gained fame because of its excellent conductivity, low toxicity, cost-effectiveness, and higher stability. The Dop casting method was utilized to fabricate V_2_C film, and it has shown a very uniform distribution on the substrate with the lower thickness (Figure 9D) [99]. 

To differentiate between the different method’s effects on thin film formation, Gogotsi et al. fabricated Ti_3_C_2_ MXene thin films by using vacuum filtration and spray-coating methods [140]. MXene thin film with a thickness of about 1 µm was fabricated utilizing the VAF, while the SCM was used for 100 nm film thickness. XRD confirmed that interlayer spacing for spray-coated Ti_3_C_2_ MXene was 17 Å, while for vacuum filtered film, it was 12.9 Å. A narrow peak at 6.8° was observed for VFM film, and after annealing this film at 150 °C, the peak shifted to 8.3°. In contrast, a broader peak at 6.1° was observed for spray-coated film, which remained constant after annealing at the same temperature. The decreased interlayer spacing of film fabricated using VAF indicates less confined water molecules present between the layers. In contrast, a higher number of water molecules are present in the case of spray-coated film. It revealed that the vacuum-coated films have higher conductivity values (Figure 9E). In another study, dip-coating and spray-coating methods were utilized to study thin film behavior [141]. However, somehow, in terms of roughness and uniformity, Ti_3_C_2_ films fabricated from both methods have shown a similar response. However, in the case of absorption spectra, the dip-coating method with 40-layer pairs of the films has shown higher absorbance than the films fabricated by the spray-coating method, which revealed that the thickness of the films fabricated by dip coating was much higher (Figure 9G).

VFM has some limitations, such as the area of the film was defined by the filter membrane used, its time consuming to obtain larger areas of thin films, and it will cost more energy than other methods. Other methods, including spray, spin, and dip coatings, produce MXene with loose flakes, affecting the conductivity of the films. Hence, the drop-casting method seems efficient for larger area films. In summary, all the methods for the fabrication of MXene thin films have shown excellent uniformity, conductivities, and a wide range of thickness with lower roughness. Somehow, the drop-casting method proved to be an easy-to-use and efficient method for getting large-scale MXene thin films. The VAF is mainly used for thicker films used in supercapacitors and batteries. MXene thin films fabricated by spin and spray-coating methods have shown lower thickness (2–300 nm) and can be utilized for solar energy applications. The contact angle and concentration of MXene solution in the film fabrication process played an essential role in comparing the quality of the fabricated thin films. In the case of SCM, the contact angle and concentration of MXene remained consistent in all bilayers, but dip or spray coating with a time variety of contact angles has been observed with rough film surfaces. Further, Ti_3_C_2_, Ti_2_C, V_2_C, Mo_2_C, or any other MXene thin films synthesized using physical or chemical methods mentioned above have shown excellent physical and chemical properties with coating adaptability on any substrate.

## 6. Applications of Thin Films of MXenes

MXene thin films owing to exceptional conductivity, flexibility, and functionality, have been used in plenty of applications, including energy storage, biomedical, catalysis, and electromagnetic interference shielding. Ease in film fabrication of different MXene materials makes it a potential candidate for various applications in batteries, solar cells, supercapacitors, sensing, separation, and electronic devices.

### 6.1. MXene Thin Films for Electromagnetic Interference Shielding 

All electronic devices that use electrical energy generate electromagnetic interference (EMI). Electromagnetic interference affects the performance of electronic devices. An increase in EMI in devices could cause substantial damage not only to devices but also it can affect human health. A shielding material with strong, defect-free, highly conductive, and electromagnetic radiation absorption properties are always required to stop this EMI effect [142]. Plenty of 2-D materials, including metals, polymer matrices, and composite materials, have been explored to prevent EMI, but their limitations inhibit their direct use in electronic devices [143]. MXene thin films played a vital role in EMI shielding because of their excellent conductivity, flexibility, and ease of binding with different polymers [144]. To obtain a higher EMI shielding effect, controlling the concentration of MXene, thickness of thin films, and conductivities is mandatory. 

**Figure 10:**
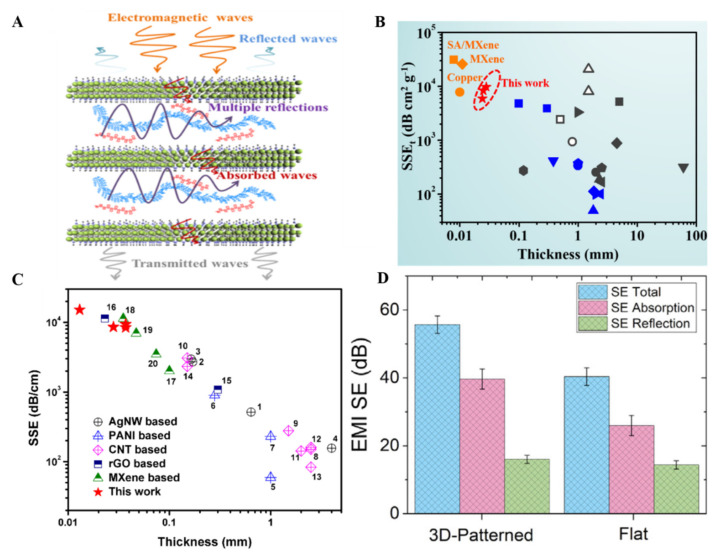
(**A**) EMI shielding mechanism uses Ti_3_C_2_ MXene thin films (reproduced with permission [68]. Copyright 2018, American Chemical Society); (**B**) Specific EMI shielding dependence on the thickness of Ti_3_C_2_ MXene thin films (reproduced with permission [146]. Copyright 2020, Elsevier); (**C**) Natural polymer doped MXene thin films for higher specific EMI shielding (reproduced with permission [147]. Copyright 2020, Elsevier); (**D**) Comparison of total EM radiation absorption and reflection for free-standing and flat MXene thin films (reproduced with permission [98]. Copyright 2018, Wiley-VCH).

Firstly, Shehzad et al. fabricated 45 μm highly flexible sodium alginate-based Ti_3_C_2_ MXene thick films with excellent EMI shielding effectiveness of about 92 decibels [145]. Ti_3_C_2_ thick film architecture and conductivity played a vital role in providing a higher shielding effect. EM waves are reflected from the surface of the first layer of MXene because electrons and the remaining waves pass through the films. The passed waves interact with MXene electrons and induce current there, which causes ohmic losses, reducing the energy of EM waves. Finally, back and forth, a reflection of these EM waves in between the layers of MXene causes their complete absorption in layers (Figure 10A). 

After pure MXene, different MXene composites have been explored for EMI shielding applications. A composite material can provide beneficial aspects over the pure MXene material. For instance, Weng et al. have used spin-spray coating layer-by-layer method to develop semitransparent MXene and carbon nanotubes-based EMI shielding material. The composite thin films have higher conductivity (130 Scm^−1^) and a higher specific shielding capacity of about 58 187 dB cm^2^ g^−1^ [107]. Similarly, polymer films utilized for the EMI shielding effect and different fillers in the polymer matrix enhanced the EMI shielding effect. In this regard, Jin et al. fabricated 27 μm thick poly (vinyl alcohol) mixed MXene films using multilayered drop-casting. These films provided higher conductivity (716 S/m) along with suitable specific EMI shielding effectiveness (9343 dB cm^2^ g^−1^) (Figure 10B) [146]. Mechanical stability and higher conductivity are required to obtain a higher EMI shielding effect. Doping of another material does improve not only the conductivity but also the mechanical properties of MXene. Multivalent aluminum ions doped MXene thin films have shown exceptional mechanical strength and blocked almost 99.99% of EM radiations. Aluminum ions provided extra stability to MXene thin films that could also resist oxidation in water [60]. Compared to synthetic polymers, naturally available polymeric materials are easy to use, and they are biodegradable with significantly lower toxicity that can be used for EMI shielding. In this regard, Liu et al. fabricated chitosan-doped Ti_3_C_2_ MXene thin films (30 µm) by using VFM to obtain an enhanced EMI shielding effect with specific EMI shielding effectiveness of about 15,153.9 ± 153 dB cm^−1^. These films were helpful in the EMI shielding effect and applied in monitoring the humidity in the air because of the exceptional conductivity of MXene thin films (Figure 10C) [147].

The primary purpose of utilizing different methods to fabricate MXene thin films for EMI shielding is to obtain a clear insight into which method is most suitable and reliable to work at an industrial scale. VAF has also been utilized to fabricate thin films of Hydroxyethylcellulose mixed with Ti_3_C_2_ MXene to obtain flexible and green EMI shielding material. The integrated electron migration in Ti_3_C_2_ MXene thin films causes the dissipation of EM waves in the form of heat [148]. The drop-casting method has also been explored to fabricate free-standing films of Ti_3_C_2_ MXene on a large scale to obtain the EMI shielding effect. About 38% increased EMI shielding was obtained with such free-standing films compared to the regular flat films. This increase was due to the 3D pattern of MXene films because various incident angles were observed after the scattering of EM waves on 3D films. Hence the higher absorption was measured for the free-standing MXene thin films (Figure 10D) [98].

**Figure 11:**
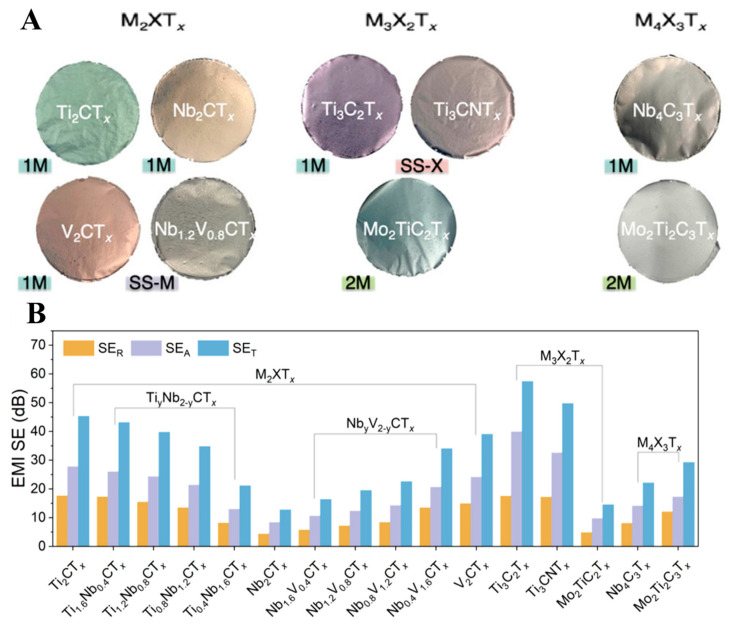
(**A**) Digital images of mono-transition metal MXenes (1M), double-transition metal MXenes (2M), Solid solution on M sites (SS-M), Solid solution on X sites (SS-X) by utilizing VFM, and (**B**) The average EMI shielding reflection, absorbance, and transmittance (SE_R_, SE_A_, and SE_T_) of variety of MXene thin films with thickness range from 5 ± 0.3 μm in the 8.2–12.4 GHz range (reproduced with permission [149], Copyright 2020, American Chemical Society).

Almost 30 different MXene materials have been explored for the EMI shielding effect. Han et al. published an excellent systematic and comparative study of 16 other MXene materials for the EMI shielding effect to study the impact of the layer structure, metal element arrangements, and elemental composition [149]. Monometal MXenes ordered double-metal MXenes, and random solid solution MXenes were synthesized in the lab. Their thin films were fabricated using spin-casting, spray coating, and VAF with thicknesses ranging from nanometers to micrometers for the EMI shielding effect (Figure 11A). A study of electricity dependence and thickness of all the films on the EMI shielding effect revealed that higher conductivity and the higher thickness could provide specific EMI shielding exceeding 20 dB. The average EM radiation absorption and reflection of all the MXene thin films were monitored. Results have shown that mono-X MXene thin films have a better EMI shielding effect than other double-X MXenes (Figure 11B). The MXene synthesis procedure, film formation method, and MXene type strongly affect the EMI shielding effect. Therefore, it is always important to control the concentration of MXene and thickness of MXene thin films for composite materials fabrication to obtain an excellent EMI shielding effect. Along with MXene, a suitable dopant also affects the electrical, mechanical, and EMI shielding properties of MXene thin films.

### 6.2. MXene Thin Films in Batteries

**Figure 12:**
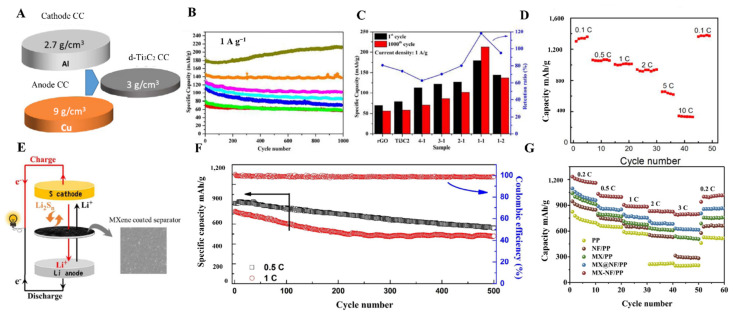
(**A**) Comparison of MXene thin film with Cu and Al foil and their densities (reproduced with permission [150]. Copyright 2018, American Chemical Society); (**B**,**C**) Cycle performance and capacity retention ratio of rGO, Ti_3_C_2_, and different composites of rGO@ Ti_3_C_2_ at 1 A g^−1^ current density after 1000 cycles (reproduced with permission [52]. Copyright 2018, American Chemical Society); (**D**) Rate performance of spray-coated Ti_3_C_2_T_x_/NiCo_2_O_4_ films (reproduced with permission [151]. Copyright 2016, Elsevier); (**E**) Schematic diagram showing the use of MXene-coated polypropylene separator; (**F**) MXene@polypropylene-based separator based improved cycling performance of the Li-S batteries at 0.5 C and 1 C rates (reproduced with permission [131]. Copyright 2016, American Chemical Society); (**G**) Comparison of rate performances of MX-NF/polypropylene cells with polypropylene, Nafion@polypropylene, MXene@polypropylene, MX@Nafion/polypropylene (reproduced with permission [152]. Copyright 2019, Elsevier).

MXene with excellent electrical conductivity (6500 S cm^−1^) can behave like a semiconductor or metallically conductive material. Electronic properties of the MXene materials can be tuned by doping with a suitable organic or inorganic nanomaterial. Owing to exceptional conductivity and capacitance (300 F cm^−3^), even in the presence of bigger cations that can be inserted and extracted into the MXene layers and have shown excellent power for the storage of multivalent cations such as Mg^2+^, Al^3+^, Ca^2+^, MXene has been widely explored for battery materials. MXene materials have been utilized as electrode materials and separators (Table 1. MXene nanosheets owing to large interlayer spacing can accommodate Li ions and can feasibly be used in Li-ion batteries. The lithium storage of MXenes depends on plenty of factors, including the synthetic approach of MXenes, M element, their surface chemistry, entire porous structure, and doped atoms. If there are F and OH groups on the surface of MXenes, it will hinder the storage of Li^+^ by blocking the transportation of Li^+^. Hence, by changing the surface chemistry of MXenes, it is possible to tune the Li^+^ storage. MXenes thin films are also used as a current collector material in batteries. In this regard, Wang et al. designed 5 µm thick films of Ti_3_C_2_ MXene as an anode material and LiFePO_4_ as a cathode material (Figure 12A) [150]. MXene thin film-based electrodes cause poor ions transport, which usually leads to loss of surface reactivity and conductivity. Ma et al. designed 3D porous Ti_3_C_2_/r-GO-based films using the VAF method to avoid such problems. These films showed excellent rate capability of 98.9 mAh g^−1^ at 4 A g^−1^ and outclass performance of 212.5 mAh g^−1^ for lithium-ion batteries without any degradation (Figure 12B,C) [52]. Meanwhile, V_2_C, in comparison with Nb_2_C and Ti_2_C, presented developed capacity, i.e., 280 mAh g^−1^. The most favorable method to tune the band gap and surface functionality of MXenes involved the introduction of heteroatoms. Inserting different heteroatoms, including Mo atoms and Co^2+^, into Mo_2_TiC_2_T_x_ and V_2_C MXenes to obtain thin films by using the VAF method guaranteed the increase in Li^2+^ storage capacity.

The fabrication method for thin films of MXene could affect the performance of Li ions storage in Li-ion batteries. For instance, Zhao et al. hybridized transition metal oxides such as Co_3_O_4_ and NiCo_2_O_4_ with Ti_3_C_2_ MXene using three different approaches, including repeated filtration, spray coating, and wet chemical method. As prepared, flexible metal oxide and MXene-based films were utilized in Li-ion batteries without any binder. The free-standing hybrid films synthesized using spray coating have shown excellent electrochemical performance with a high reversible capacity of about 1330 mAh g^−1^ at 0.1 C (Figure 12D) [151].

In Li-S batteries, MXene has played an essential role as a separator [153]. Plenty of materials, including carbon nanotubes, graphene/graphene oxide, TiO_2_, and other 2-D nanomaterials, have been used as a separator to suppress the migration of polysulfides [136,154,155]. MXene coatings by using VFM have also been used on polypropylene membranes as a separator in Li-S batteries, and it causes a significant improvement in cycling stability and rate capability of Li-S cells (Figure 12E,F) [131]. Similarly, Wang et al. modified the polypropylene separator with Ti_3_C_2_ MXene and Nafion composite to inhibit the shuttle effect. VFM has been utilized to obtain a layer-by-layer modification of Nafion with MXene material. This composite material provides excellent conductivity and suitable cycling stability and causes effective transport of Li ions with highly enhanced initial capacity and rate performance compared to other materials (Figure 12G) [152]. Suitable charge capacity and excellent stability are crucial requirements for a suitable battery. MXene-derived derivatives also played an essential role as a separator in batteries. TiN@C sheets derived from MXene acted as a separator in Li-S batteries, and an excellent initial discharge capacity at 0.1 C (1490.2 mAh g^−1^) was obtained by VFM [156]. MXene materials proved efficient in preparing stable, long life, and higher charge capacity batteries because their exceptional electrical, physical, and chemical properties and surface modification could bring more to the battery world.

### 6.3. MXene Thin Films in Sensors

Sensing devices will be a great need in the near future in industries, homes, hospitals, and most places. Plenty of materials, including graphene, metal nanoparticles, carbon nanotubes, and black phosphorous, have been explored for gas sensing, body motion sensing, heavy metal ion detection, and organic moieties sensing. Materials owing high conductivity, ease in functionalization, flexibility, and long-term stability are essential for next-generation self-powered sensors. MXene thin films with all the aforementioned properties have been explored for various sensors. Zhang et al. used layer-by-layer self-assembly process to fabric MXene/black phosphorus thin films applied in pressure sensors with o 77.61 kPa^−1^ pressure sensitivity at 0.45 MPa elastic modulus. Furthermore, this flexible sensor has shown a fast response time and monitors human heart motion [157]. 

**Figure 13:**
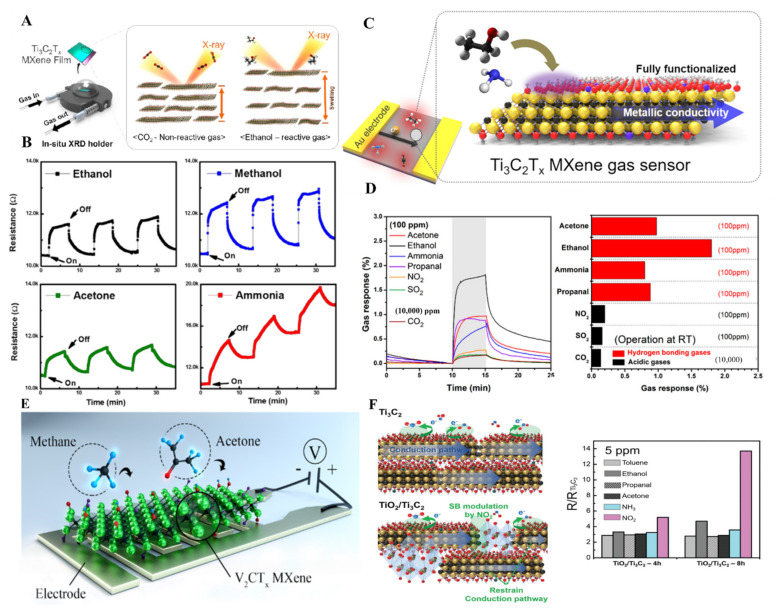
(**A**) Schematics of in situ XRD analysis of Ti_3_C_2_ thin films sensing mechanism, (reproduced with permission [158]. Copyright 2019, American Chemical Society); (**B**) Gas-sensing results of thin Ti_3_C_2_ films-based gas sensors in response to 100 ppm ethanol, methanol, ammonia, and acetone at room temperature (reproduced with permission [160]. Copyright 2017, American Chemical Society); (**C**) Schematic of chemiresistive Ti_3_C_2_ thin films-based gas sensor; (**D**) Resistance change and maximal resistance variation in Ti_3_C_2_ MXene thin film gas sensor upon exposure to 100 ppm of acetone, ethanol, ammonia, propanol, NO_2_, SO_2_, and 10,000 ppm of CO_2_ at room temperature, (reproduced with permission [161]. Copyright 2018, American Chemical Society); (**E**) Schematic showing gas-sensing mechanism of V_2_CTx MXene thin film, (reproduced with permission [99]. Copyright 2019, American Chemical Society); (**F**) Ti_3_C_2_ and TiO_2_/Ti_3_C_2_ thin film gas-sensing mechanism toward NO_2_ gas and comparison of enhancement factor of gas response for Ti_3_C_2_ and TiO_2_/Ti_3_C_2_ film at five ppm gas concentration (reproduced with permission [163]. Copyright 2020, Wiley-VCH).

MXene thin films, because of their 2-D layer structure and ease in surface modification, have played an important role in gas sensing. Ti_3_C_2_T_x_ owing to ease in surface termination, has been tested for NH_3_ sensing. To check the changes happening in MXene thin films during the sensing mechanism, Koh et al. have performed an in situ X-ray study of Ti_3_C_2_ MXene thin films fabricated using VAF upon CO_2_ and ethanol sensing. An X-ray study revealed that MXene thin films displayed dynamic and selective swelling upon interaction with ethanol over CO_2_ in the presence of NaOH (Figure 13A) [158]. Similarly, the insertion of Na ions in Ti_3_C_2_ MXene thin films prepared by the dip-coating method improved the humidity and gas-sensing properties of thin film by increasing the oxygen-fluorine groups on the surface [159]. To obtain a clear insight into the mechanism of gas sensing, Lee et al. fabricated Ti_3_C_2_T_x_ thin films by drop-casting method and tested for sensing of ammonia, ethanol, methanol, and acetone gas (100 ppm) at room temperature by tuning the electrical properties of MXene thin films. Due to the lack of F group on the surface, Ti_3_C_2_T_x_ MXene thin films have shown higher affinity toward ammonia gas molecule and lowest toward acetone gas in a chemiresistive sensor (Figure 13B) [160]. The basic requirements for the highly sensitive sensor are low electrical noise and strong signal production, and development in MXene thin film-based sensors fulfill these requirements. Basically, gas adsorption on MXene thin film effect charge carrier transport, resulting in the change in resistance usually measured. In this regard, Kim et al. fabricated pure Ti_3_C_2_T_x_ MXene thin films by VFM for acetone, ammonia, ethanol, propanol, NO_2_, SO_2,_ and CO_2_ gas sensing [161]. The MXene-based sensor has shown a change in resistance under exposure to 100 ppm of all the mentioned gases, which is a suitable agreement that on gas adsorption, the charge carrier transportation gets disturbed. The Ti_3_C_2_T_x_-based sensor has shown a suitable response to ethanol with a limit of detection of 50 ppb to 1000 ppm because of hydrogen bonding. It has demonstrated the most negligible response to NO_2_, SO_2,_ and CO_2_ gases (Figure 13C,D). The thickness of the MXene films also affected the gas-sensing mechanism because of the available active sites. MXene 3D porous structures based on the thin film by using the electrospinning method have also been used as a gas sensor for acetone, ethanol, methanol, ammonia, toluene, water vapor, and NO_2_ sensing. Polar gases obtained an extraordinary response because of the polar surface of MXene thin films, while the non-polar gases did not show any significant change in resistance on interaction with MXene thin films [162].

Regardless of the higher conductivity and exceptional sensing properties of Ti_3_C_2,_ other MXene materials have also shown suitable sensing abilities because of their non-polar nature. For instance, Lee et al. prepared single/few layers of vanadium carbide MXene thin films (V_2_CT_x_) on polyimide film by VFM and used them as a chemiresistive sensor for polar (hydrogen) and non-polar (methane) gas sensing with 2 and 25 ppm limit of detection (Figure 13E) [99]. Similarly, molybdenum carbide (Mo_2_C) thin film fabricated by VFM has also been used for NH_3_ and NO_2_ gas sensing with gases concentration ranging from 0.125 to 5 ppm [164]. Furthermore, except for pure MXene, mild reduction in MXene also proved efficient for gas-sensing applications. As in the case of Ti_3_C_2_, control reduction in Ti_3_C_2_ can produce TiO_2,_ which can be further used as a composite TiO_2_/Ti_3_C_2_ material for chemiresistive gas sensors. VFM produced TiO_2_/Ti_3_C_2_ MXene film treated with an alkaline solution and used for selective sensing of NO_2_ gas with a 125 ppb limit of detection (Figure 13F) [163]. In conclusion, although all the MXene thin film fabrication seems great for gas sensing as compared to drop-casting, vacuum-filtration, and dip-coating methods, the electrospinning method can fabricate thinner MXene films that could lead to higher active surface area and efficient gas sensing. Alkali treatment of all the MXene films before gas sensing contributes to the gas sensing mechanism. It is possible to functionalize MXene thin films surface with different chelating agents and polymers to obtain selective gas sensing that can be further used in industries.

### 6.4. Mxene Thin Films in Solar Cells

To overcome future energy crises and global warming, researchers are inclined to progress with renewable energy sources. Among various sources, the solar energy source is considered a competitive candidate to deal with future clean energy demands. For this purpose, multiple efforts have been made to develop efficient and stable solar cells by introducing novel materials. Two-dimensional Mxenes have recently attracted increasing attention in solar cells application due to their high metallic conductivity, superior carrier mobility, high transparency, and tunable work function [50]. In this review, we have highlighted the use of MXenes thin films prepared by different methods for solar cell application. 

Mxenes have been considered a promising electron transport layer (ETL) or a dopant in ETL for efficient and stable perovskite solar cells. Yang et al., spin-coated Ti_3_C_2_ Mxene doped SnO_2_ as an ETL in n-i-p perovskite solar cell (PSC) [111]. The resulting ETL provides superior charge transfer paths, boosts electron extraction, and minimizes the electron transfer resistance at the perovskite and ETL interface. The SnO_2_-Ti_3_C_2_ spin-coated ETL enhanced the power conversion efficiency (PCE) from 17.23% to 18.34%. Wang et al. modified the SnO_2_ with Ti_3_C_2_ Mxene spin coated over the FTO, promoting superior mobility, higher charge transfer ability of SnO_2,_ and strong interface interaction [165]. Further, the modification of SnO_2_ with Mxene enabled the preferable growth platform for the perovskite film with reduced trap densities. As a result, an enhanced PCE of 20.65% was achieved with negligible hysteresis. Lin Yang et al. treated the spin-coated Ti_3_C_2_ Mxene with UV-ozone, improved electron transfer at the ETL/perovskite interface, and suppressed the recombination as well [115]. The devices fabricated based on UVO-treated Ti_3_C_2_ result in 17.17% champion PCE compared to UVO untreated devices with only 5%. PCE. Saranin et al., spin-coated Mxene doped PC_61_BM ETL in p-i-n PSCs to modify the work function and band alignment of PC_61_BM relative to the perovskite layer [118]. The Mxene-modified device achieved 19% PCE with improved stabilized power output compared to the reference device. Agresti et al. tuned the perovskite film work function from 4.72 to 4.37 eV by spin coating Mxene doped MAPbI_3_. As a result, 26.5% improvement in PCE was achieved after simultaneously spin coating Ti_3_C_2_ as an ETL with Mxene doped MAPbI_3_ [114].

**Figure 14:**
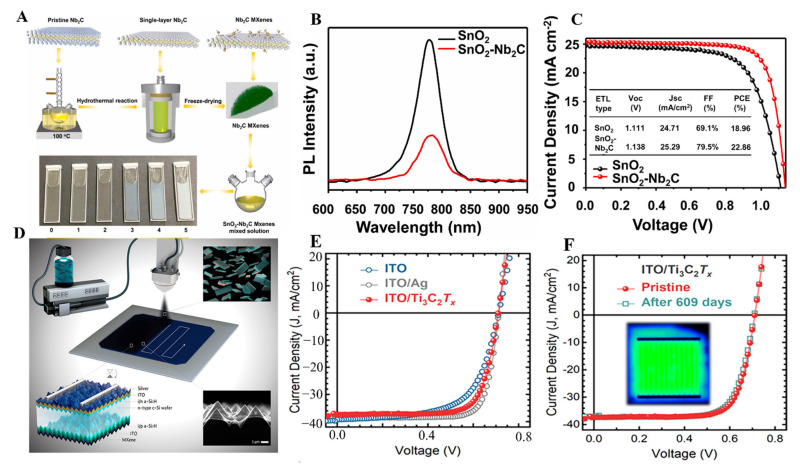
(**A**) The preparation process of SnO_2_-Nb_2_C MXene solution; (**B**) Photoluminescence spectra of SnO_2_ and SnO_2_-Nb_2_C MXene; (**C**) J-V curves, and photoconversion efficiency of SnO_2_ and SnO_2_-Nb_2_C MXene thin films fabricated by an SCM, reproduced with permission [124]. Copyright 2020, American Chemical Society); (**D**) Schematic representation of the automated spraying apparatus of large-scale deposition of Ti_3_C_2_T_x_ flakes as the back electrode for SHJ solar cells, Insets: (Bottom-left) Corresponding layer-by-layer structure of an SHJ solar cell. (Bottom-right) Tilted top-view SEM micrograph of the Ti_3_C_2_T_x_ flakes covering the ITO-coated pyramidal textured surface of SHJ solar cells; (**E**) JV curves of ITO, ITO/Ag, and ITO/MXene thin films; (**F**) JV curves after 609 days of a device fabricated (reproduced with permission [166]. Copyright 2020, American Chemical Society).

Furthermore, the Nb_2_C-SnO_2_ ETL spin coated over the ITO enables a larger SnO_2_ grain size and provides higher quality and superior crystalline perovskite film [124]. The detailed Nb_2_C-SnO_2_ ETL process is demonstrated in (Figure 14A). The high-angle annular dark-field scanning transmission electron microscopy (HAADF-STEM) confirmed that the doping of the Nb_2_C MXene increased the lattice spacing facets of SnO_2_, which improves the surface energy, roughness, and defects in SnO_2_-Nb_2_C-based ETL. The lattice fringes correspond to (101) (110) SnO_2_ with the spacing of 0.320 (B_1_) and 0.240 nm (B_2_). Similarly, the incorporation of Nb_2_C in SnO_2_ changes the lattice spacing to 0.253 (C_1_) and 0.330 (C_2_) nm corresponding to (101) and (110) SnO_2_. Moreover, the Nb_2_C introduction enhances the grain size of SnO_2_ from 179 nm to 256 nm. The Nb_2_C-SnO_2_ ETL results in PCE of 22.86% compared to the control device with 18.96%.

Furthermore, the PL spectra indicate that the addition of Nb_2_C in SnO_2_ had a lower PL emission that confirms the reduced recombination and enhanced photogenerated carrier generation (Figure 14B). Subsequently, the devices fabricated on SnO_2_ and Nb_2_C SnO_2_ had a PCE of 18.96% and 22.86%, respectively (Figure 14C). The device stability was tested under 40–60% humidity. The Nb_2_C-SnO_2_ ETL retained 98% of the initial PCE, while the SnO_2_ ETL retained only 85%. This indicates the effectiveness of Nb_2_C doping in SnO_2_ for stable PSCs.

Besides ETL, MXenes are also a suitable material as a dopant in HTL. Hou et al., spin-coated MXene doped PEDOT: PSS results in more charge transfer channels between PEDOT nanocrystals in organic solar cells [167]. The addition of MXene transformed the PEDOT from coil structure to linear coil structure, leading to enhanced electrical conductivity. The MXene doped PEDOT: PSS spin-coated film achieved 11.02% PCE compared to 9.72% for the controlled device.

Spray coating has been considered a well-known method for fabricating dense, homogeneous, and uniform MXene thin films. Cao et al. spray-coated MXene paste over the MXene photoanode as a back electrode [119]. The sprayed MXene electrode was hot-pressed to create a seamless contact between perovskite and Ti_3_C_2_. The device was prepared based on the MXenes electrode and had a PCE of 13.83%, 27% higher than the device designed on a carbon-based electrode. Recently, spray-coated MXenes electrodes were fabricated to replace Ag for SHJSC at an Industrial-scale [166]. The fabricated MXene contacts had a high uniform coverage over the typical micron-scale pyramidal textured backside of SHJSC. The MXene spray-coated electrode results in more than 20% PCE over medium (4.2 cm^2^) and large (243 cm^2^) cell areas with 99% PCE retention for over 600 days in an ambient environment. The schematic of the spray-coating process of the MXene electrode is demonstrated in (Figure 14D). Although spray-coating enables uniform and fast coating over a large area, this becomes challenging in the case of complex geometries such as random-pyramidal textured SHJSC. Therefore, the uniformity of the surface coverage has been determined by scanning electron microscopy. The cross-section SEM graphs indicate the complete range of MXene over the facts of backside pyramids. To analyze the optimum MXene rear contact thickness on SHJ cells, an array of devices was fabricated with a cell area of 4.2 cm^2^ by spraying MXene dispersion in DI water (Figure 14D). The MXene spray-coating cycles varied from 40 to 260. The fabricated devices based on different thicknesses showed 140 cycles as an optimum value with the highest PCE of 19.8%. Upon increasing the spraying cycles to 160, the PCE dropped to 19.3%. Moreover, the further increase in spraying cycles to 260 causes a further reduction in PCE to 18.9%. J–V curves of devices with different rear electrode stacks (Figure 14E). The presence of metallic contact to the rear ITO has a higher PCE and FF than without contacted cells. The reason is that Ag-connected cells provide better back reflection than MXene-contacted cells due to the high absorption cross-section of Ti_3_C_2_ films. Thus, the NIR response of the MXene-based device is slightly less than the Ag-based device revealed by EQE measurements. The stability of the MXene-based devices was tested after keeping them in an ambient environment for more than 20 months (Figure 14F). The PCE of the device dropped to 19.5% after such a long time duration. Ag-based devices remained unchanged for a long time, but such stability for MXene-contacted devices is notable considering the oxidation sensitivity of MXenes.

During the past few years, spin-coated MXene thin films have been developing progress in solar cell research. However, spray-coating can be a viable option to fabricate MXenes films on an industrial scale. Further, other methods, such as dip coating, doctor blade coating, and inkjet printer films, can scale up the MXene thin film preparation for solar cell application.

### 6.5. Miscellaneous Applications

**Figure 15:**
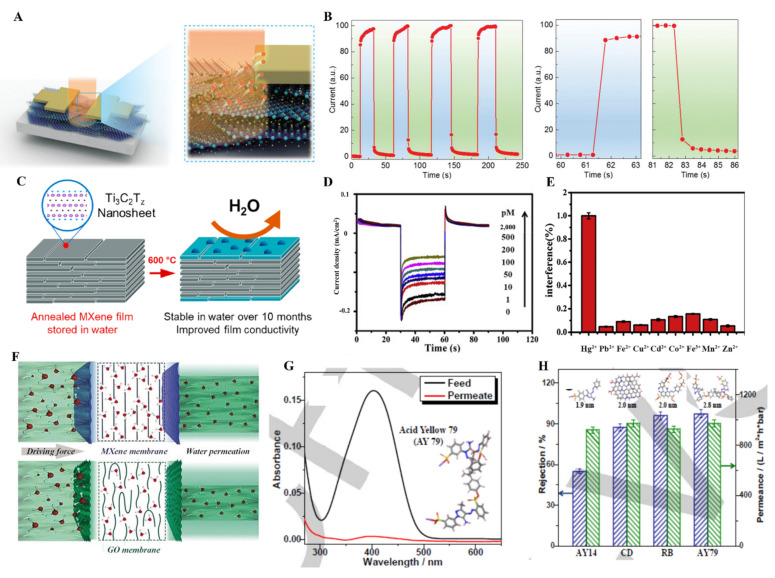
(**A**) Schematics of plasmon-assisted hot electron migration toward gold electrode in Mo_2_CT_x_-based photodetector, (reproduced with permission [51]. Copyright 2019, Wiley-VCH); (**B**) Increase and decrease the cycle of photocurrent upon switching the light ON and OFF in the case of Mo_2_CT_x_ thin film-based photodetector (reproduced with permission [51]. Copyright 2019, Wiley-VCH); (**C**) Schematic of Ti_3_C_2_T_x_ MXene thin films annealed at 600 °C and stored in water for 10 months. Only the upper surface is oxidized and protects the inner layers from oxidation (reproduced with permission [168]. Copyright 2020, American Chemical Society); (**D**) Change in photocurrent upon testing Ti_3_C_2_T_x_@BiVO_4_ thin film-based photoelectrochemical sensor against the different concentrations of Hg^2+^ (reproduced with permission [171]. Copyright 2020, Elsevier); (**E**) Selectivity of Ti_3_C_2_T_x_@BiVO_4_ thin film-based photoelectrochemical sensor for Hg^2+^ in the presence of other metal ions (reproduced with permission [171]. Copyright 2020, Elsevier); (**F**) Schematics of the mechanism of separation of organic moieties by using Ti_3_C_2_T_x_ membrane, (reproduced with permission [172]. Copyright 2018, Wiley-VCH); (**G**) UV visible absorption measurements for AY-79 dye in propanol before and after filtration by using MXene membrane; and (**H**) Different dye molecules separated by using MXene filter (reproduced with permission [172]. Copyright 2018, Wiley-VCH).

MXenes owing to exceptional optical and photothermal properties have been explored for photodetectors. Photodetectors convert incident electromagnetic radiation in the UV, visible or infrared region into electrical signals. Photodetectors usually do not require pure metallic materials or metal-like conductivities; hence Ti_3_C_2_ may not be an excellent choice for photodetectors. Among different MXene materials, including Mo_2_CT_x_, Ti_3_C_2_T_x_, Nb_2_CT_x_, T_2_CT_x_, and V_2_CT_x,_ Velusamy et al. tested Mo_2_CT_x_ thin films for photodetection purposes. Parallel arrays of Mo_2_CT_x_ thin film photodetectors were deposited on a paper substrate. The photodetectors’ responsivity and detectivity reached about 9 AW−1 and ≈5 × 1011 Jones, almost 18,000 and 1200 times higher than already reported graphene-based photodetectors. Such a high photoresponse in the visible region is because of plasmon-assisted hot carriers (Figure 13A) [51]. Because of the movement of these hot electrons, an increase and decrease in photocurrent were observed by just switching the light ON and OFF, respectively (Figure 15B). Although it has been observed that Ti_3_C_2_ MXene is not stable in an open environment and can be oxidized to TiO_2_ but Ti_3_C_2_ still MXene thin films have shown extraordinary performance in acting as protective coatings. VFM is utilized for the fabrication of Ti_3_C_2_ MXene thin films that, upon annealing at a high temperature (600 °C) in an argon environment, the outer surface of thin films oxidized to TiO_2_, which acts as a protective layer and can stop the further oxidation of sandwiched films and act as a perfect oxidation-resistant coating of the materials for almost 10 months (Figure 15C) [168].

Heavy metal ions detection is a globally concerning research topic because of their significant toxicity to humans. Plenty of 2-D materials and thin films have been widely explored to detect heavy metal ions because of their high surface area and functional surfaces [169]. The electrostatic interactions and absorption involved in the detection of heavy metal ions by 2-D materials thin films. Detection of multiple heavy metal ions by utilizing a single 2-D material-based thin film was not discovered because of the non-availability of higher surface area, water flow, and favorable surface interaction with heavy metal ions. To gain such properties, Xie et al. fabricated reduced graphene oxide-based Ti_3_C_2_T_x_ thin films by VFM for pressure-free detection of multiple heavy metal ions, including HCrO^−4^, AuCl_4_^−^, PdCl_4_^−2^, and Ag^+^ [170]. Reduced graphene oxide was used to mitigate the restacking Ti_3_C_2_T_x_ thin films. The most favorable mechanism for the detection of heavy metal ions by using Ti_3_C_2_T_x_ thin films is considered to be the reduction in these ions to their respective non-toxic form as in the case of HCrO^−4^, Ti_3_C_2_T_x_ provided free electrons and converted Cr(IV) to Cr(III), and similarly, Au, Pd, and Ag metals were obtained after their ions reduction. Moreover, Jiang et al. fabricated a Ti_3_C_2_T_x_@BiVO_4_ thin film-based photoelectrochemical sensor for selective detection of Hg^2+^. This sensor displayed a wide detection range from 1 pM to 2 nM (Figure 15D) [171]. Selectivity is another important factor considered while designing such sensors, and the Ti_3_C_2_T_x_@BiVO_4_ thin film-based sensor has shown excellent selectivity toward Hg^2+^ in the presence of other ions (Figure 15E).

MXene thin films, due to their extraordinary small thickness, have been explored to filter unrequired guests. Wang et al. utilized VFM to acquire regular and lamellar membrane of Ti_3_C_2_T_x_. The rigidity of Ti_3_C_2_Tx results in the formation of an ordered 2 nm channel, and this membrane is applied to separate dye molecules from water (Figure 15F) [172]. The performance of such membrane was tested using AY-79 membrane with water and passing through this filter channel of MXene. The UV visible spectra were measured, showing that after passing through the membrane, the apparent peak of AY-79 was diminished because of molecular solid rejection. Similarly, other dyes with different sizes were also filtered by using a 2 nm channel of these MXene films (Figure 15G,H). The thickness of the MXene films played an essential role in detecting heavy metal ions. Different methods can provide a different thickness of thin films and can be used to tune the thickness of films. In the case of heavy metal ion detection, an optimized thickness is necessary to achieve the lowest possible detection limit and suitable sensitivity toward a single metal detection [173].

In recent years, along with diversity in the class of MXene thin films, a more comprehensive range of applicability has been observed in different fields, including sensors, photodetectors, motors, energy storage, catalysis, and separation. Few applications required specific thickness of the films, possibly obtained by utilizing different methods. MXene thin films have been widely explored in lab-based applications, but still, an improvement is required for industrial scale. Further, doping of MXene with other elements could enhance their applicability in energy storage, sensing, and biomedical field [174]. Lastly, advanced electron microscopic studies of thin films of MXene materials could open a new door to surface functionalization and modification of such a novel material [175].

## 7. Conclusion and Outlook

MXenes thin films have been explored widely because of their unique physical, chemical and electrical properties. This review explained MXenes thin film fabrication strategies, including vacuum filtration, electrodeposition, spin, spray, dip coatings, and physical deposition methods. Further, a comparative study on these methods explained well; modification or combination could further develop MXene thin film fabrication strategies. In addition, a suitable summary of progress in MXene thin film applications in electromagnetic interference shielding, solar cells, batteries, sensing, and separation is reviewed. Although MXenes thin films have been used in every aspect of science but still by improving their fabrication strategies, surface modifications, and doping chemistry, these thin films can be used in undiscovered applications, i.e., practical applications at the industrial level. By keeping these things in mind, a few things need to be considered in the future to develop the MXene thin films and their application in different fields.

Almost 30 MXene materials have been discovered, and all have not been converted into thin films, which limits the applicability of these MXene materials. Considering fabrication methods of thin films of MXenes, it is well known that spray, spin, dip-coating methods, and VAF have been studied well. Still, less attention has been devoted to fully understanding the MXene thin film synthesis mechanisms involved in electrodeposition methods and chemical-physical vapor deposition. The thickness of MXene thin films fabricated by the aforementioned methods can be tuned from the micro to the nano level. Still, more development is required to fabricate tens of nanometers thin films.

Another aspect is that MXene thin films can be easily oxidized in a humid environment, which affects the durability of the MXene films. By effectively using chemical grafting methods to modify the edges and defects of MXene thin films, oxidation can be reduced to a minimal level. In addition, lateral cross-linking of MXenes thin films and strategies to control the fabrication of vertically aligned pores could open new doors for development in electrochemical applications. In summary, the controllability, reproducibility, and robustness of the MXene-based thin film strategies need further development. 

Furthermore, MXene thin films used for separation and gas sensing should be studied well theoretically using density functional theory and other simulation software to obtain a clear insight into the mechanism. In recent years, along with diversity in the class of MXene thin films, a more comprehensive range of applicability has been observed in different fields, including sensors, photodetectors, motors, energy storage, catalysis, and separation. Few applications required specific thickness of the films, possibly obtained by utilizing different methods. MXene thin films have been widely explored in lab-based applications, but still, an improvement is required for industrial scale. These methods are being used at the lab scale for the fabrication of MXene films for a variety of applications, but to achieve industrial-scale applications still, more fabrication technologies need to be explored.

Thin films based on other 2-D materials such as gold nanoparticles, graphene, carbon nanotubes, and black phosphorous gained tremendous attention but difficulty in thin film fabrication and unsatisfying stripping strategies limit their use for advanced applications. In contrast, solution-based procession and cost-effective, large-scale production of MXene-based thin films are favorable for a wide range of applications [51,166,172,176]. MXene thin films with exceptional physical and chemical properties can be further explored to achieve unique properties and outstanding performance. We believe that continuous development from diverse fields of chemistry, material science, and physics will further revolutionize the development of MXene thin films for advanced technologies at the lab and industrial scale.

## Figures and Tables

**Table 1:** Summary of MXene materials films synthesized using different methods and their applications in energy storage.

MXene Material	Method	Performance	Applications	Refs
			Solar Cells	
Ti_3_C_2_T_x_ QDs and SnO_2_	Spin coating	ETL power conversion efficiency (PCE) 23%	Perovskite solar cell	[110]
Ti_3_C_2_T_x_ and SnO_2_	Spin coating	ETL PCE 18.34%	Perovskite solar cell	[111]
Oxidized Ti_3_C_2_T_x_	Spin coating	ETL PCE 18.29%	Perovskite solar cell	[112]
Ti_3_C_2_T_x_	Spin coating	Additive PCE 17.41%	Perovskite solar cell	[113]
Ti_3_C_2_T_x_	Spin coating	Functional group tuning PCE 26%	Perovskite solar cell	[114]
Ti_3_C_2_T_x_	Spin coating	ETL PCE 17.17%	Heterojunction Perovskite solar cell	[115]
mTiO2-Ti_3_C_2_T_x_ QDs-CH_3_NH_3_PbX_3_	Spin coating	Active layer PCE 21.64%	Perovskite solar cell	[116]
Ti_3_C_2_T_x_ and TiO_2_	Spin coating	ETL PCE 2.81%	Cs2AgBiBr_6_ double-PSCs	[117]
Ti_3_C_2_T_x_+CH_3_NH_3_PbX_3_ and Ti_3_C_2_T_x_+PCMB	Spin coating	Active layer PCE 19.2%	NiO-based inverted perovskite solar cell	[118]
Ti_3_C_2_T_x_	Spin coating	HTM PCE 13.83%	noble-metal-free perovskite solar cell	[119]
Ti_3_C_2_T_x_+CNTs+ carbon paste	Slurry	Electrode PCE 7.09%	all-inorganic Perovskite solar cell	[120]
Ti_3_C_2_T_x_/PEDOT: PSS	Spin coating	Anode PCE 17.26%	Non-fullerene organic solar cell	[121]
Ti_3_C_2_T_x_+SWCNTs	Slurry	ETL PCE 21%	Perovskite solar cell	[122]
Plasma oxidized Ti_3_C_2_T_x_	Spin coating	ETL PCE 18.9%	Perovskite solar cell	[123]
Nb_2_C@SnO_2_	Spin coating	ETL PCE 22.86%	Perovskite solar cell	[124]
Ti_3_C_2_T_x_@n^+^-Si	Drop casting	PCE 11.5%	Silicon solar cell	[125]
			Batteries and Supercapacitors	
MXene/1T-2H MoS_2_-C-S	Hydrothermal annealing	Cathode1194.7 mAh g^−1^ at 0.1 C	Li-S batteries	[126]
Ti_2_C	Chemical etching	Anode 225 mAh g^−1^ at 1C	Li-ion batteries	[127]
Silicon@Ti_3_C_2_T_x_	Vacuum Filtration	Anode 2118 mAh·g^−1^ at 200 mA·g^−1^	Li-ion batteries	[128]
V_2_C and Nb_2_C	Chemical etching	Anode 260 mAhg^−1^ at 1C	Li-ion batteries	[129]
SnO_2_@Ti_3_C_2_T_x_	Atomic layer deposition	Anode 843 mAhg^−1^	Li-ion batteries	[130]
Ti_3_C_2_T_x_	Filtration	Separator 495 mAh g^−1^ at 1C	Li-S batteries	[131]
Ti_3_C_2_T_x_	Filtration	Separator 820 mAh/g at the current of 0.5 A/g	Li-S batteries	[132]
Vanadium carbide (V_4_C_3_)	Coating	Electrode-specific capacitance 330 F/g at 5 mV/s	Supercapacitor	[133]
Vanadium carbide (V_2_C)	Rolling between membranes for film fabrication	Electrode-specific capacitance 487 F/g	Supercapacitor	[134]
Ti_3_C_2_T_x_ and porous Vanadium nitride/carbon	Vacuum filtration	Negative electrode-specific capacitance 105 F/g at 1 A/g	Asymmetric supercapacitors	[135]
Ti_3_C_2_T_x_	Vacuum filtration	Electrode areal capacitance 71.16 mF cm^−2^	Micro-supercapacitor	[61]
Ti_3_C_2_T_x_/carbon nanotubes (CNTs)	Vacuum filtration	Electrode capacitance 300 F g^−1^ at 1 A g^−1^	Supercapacitor	[136]
Polystyrene/Ti_3_C_2_T_x_	Vacuum filtration	Gravimetric the capacitance of 506 F g^−1^ at 0.5 A g^−1^	Supercapacitor	[137]
Ti_3_C_2_T_x_	Freeze tape casting	Specific capacitance 222.9 F g^−1^	Supercapacitor	[138]
Nanoporous Ti_3_C_2_T_x_	Tablet machine pressing	Volumetric capacitance 932 F cm^−3^	Supercapacitor	[139]
Freeze-and-thaw Ti_3_C_2_T_x_	Vacuum filtration	Volumetric capacitance 591 F cm^−3^	Micro-supercapacitors	[66]
